# Transcriptome analysis of immune cells from Behçet’s syndrome patients: the importance of IL-17-producing cells and antigen-presenting cells in the pathogenesis of Behçet’s syndrome

**DOI:** 10.1186/s13075-022-02867-x

**Published:** 2022-08-08

**Authors:** Mai Okubo, Shuji Sumitomo, Yumi Tsuchida, Yasuo Nagafuchi, Yusuke Takeshima, Haruyuki Yanaoka, Harumi Shirai, Satomi Kobayashi, Yusuke Sugimori, Junko Maeda, Hiroaki Hatano, Yukiko Iwasaki, Hirofumi Shoda, Tomohisa Okamura, Kazuhiko Yamamoto, Mineto Ota, Keishi Fujio

**Affiliations:** 1 Department of Allergy and Rheumatology, Graduate School of Medicine, The University of Tokyo, 7-3-1 Hongo, Bunkyo-ku, Tokyo, 113-8655 Japan; 2 Department of Functional Genomics and Immunological Diseases, Graduate School of Medicine, The University of Tokyo, 7-3-1 Hongo, Bunkyo-ku, Tokyo, 113-8655 Japan; 3 Laboratory for Autoimmune Diseases, RIKEN Center for Integrative Medical Sciences, 1-7-22 Suehiro-cho, Tsurumi-ku, Yokohama, Kanagawa 230-0045 Japan

**Keywords:** Behçet’s syndrome, Transcriptome, Interleukin-17, YBX3, Antigen-presenting cells

## Abstract

**Background:**

Behçet’s syndrome (BS) is an immune-mediated disease characterized by recurrent oral ulcers, genital ulcers, uveitis, and skin symptoms. HLA-B51, as well as other genetic polymorphisms, has been reported to be associated with BS; however, the pathogenesis of BS and its relationship to genetic risk factors still remain unclear. To address these points, we performed immunophenotyping and transcriptome analysis of immune cells from BS patients and healthy donors.

**Methods:**

ImmuNexUT is a comprehensive database consisting of RNA sequencing data and eQTL database of immune cell subsets from patients with immune-mediated diseases and healthy donors, and flow cytometry data and transcriptome data from 23 BS patients and 28 healthy donors from the ImmuNexUT study were utilized for this study. Differential gene expression analysis and weighted gene co-expression network analysis (WGCNA) were performed to identify genes associated with BS and clinical features of BS. eQTL database was used to assess the relationship between genetic risk factors of BS with those genes.

**Results:**

The frequency of Th17 cells was increased in BS patients, and transcriptome analysis of Th17 cells suggested the activation of the NFκB pathway in Th17 cells of BS patients. Next, WGCNA was used to group genes into modules with similar expression patterns in each subset. Modules of antigen-presenting cells were associated with BS, and pathway analysis suggested the activation of antigen-presenting cells of BS patients. Further examination of genes in BS-associated modules indicated that the expression of *YBX3*, a member of a plasmacytoid dendritic cell (pDC) gene module associated with BS, is influenced by a BS risk polymorphism, rs2617170, in pDCs, suggesting that *YBX3* may be a key molecule connecting genetic risk factors of BS with disease pathogenesis. Furthermore, pathway analysis of modules associated with HLA-B51 indicated that the association of IL-17-associated pathways in memory CD8^+^ T cells with HLA-B51; therefore, IL-17-producing CD8^+^ T cells, Tc17 cells, may play a critical role in BS.

**Conclusions:**

Various cells including CD4^+^ T cells, CD8^+^ T cells, and antigen-presenting cells are important in the pathogenesis of BS. Tc17 cells and *YBX3* may be potential therapeutic targets in BS.

**Supplementary Information:**

The online version contains supplementary material available at 10.1186/s13075-022-02867-x.

## Background

Behçet’s syndrome (BS) is an autoimmune disease characterized by recurrent oral aphthous ulcers, genital ulcers, uveitis, and skin symptoms, such as pustular folliculitis and erythema nodosum. Some patients may also develop arthritis, epididymitis, vascular lesions, central nervous system lesions, and gastrointestinal lesions similar to inflammatory bowel disease. The clinical course is characterized by repeated inflammatory attacks with periods of exacerbation and remission. Mucocutaneous manifestations are often self-limiting, but uveitis, intestinal, vascular, or central nervous system diseases are often intractable and severe, impairing the patient’s quality of life. Various immunomodulatory or immunosuppressive drugs are used for the treatment of BS. Mild symptoms may be managed with topical treatment or colchicine, but more severe symptoms may require systemic steroids, immunosuppressants such as cyclosporine and methotrexate, and tumor necrosis factor (TNF)-α inhibitors [1]. However, the current treatment of BS is far from perfect, and many patients have impaired quality of life due to symptoms of BS or due to side effects of treatment [2].

Various immune cells have been implicated in the pathogenesis of BS. BS lesions such as pustular folliculitis are characterized by infiltration of neutrophils [3], suggesting a role for the innate immune system in the pathogenesis of BS. T cells are also seen in BS lesions, and an expansion of Th1 and Th17 cells has been reported in BS patients [4], suggesting a role for the adaptive immune system in the pathogenesis of BS.

BS is considered a multifactorial disease, and both genetic factors and environmental factors are involved in its pathogenesis. Among genetic factors, HLA-B51 has been strongly associated with BS in studies from various ethnic groups. In addition, BS disease-susceptible single nucleotide polymorphisms (SNPs) have been identified in or near *IL23R*, *IL12RB2*, *IL10*, *STAT4*, *IL12A*, *CCR1*, *CCR3*, *KLRC4*, *ERAP1, IL1B*, *IRF8*, and *IFNGR1* [5–10]; however, the exact mechanism by which those SNPs contribute to disease pathogenesis and the cell types where they exert their function have not been elucidated.

In recent years, transcriptome analyses of samples from various connective tissue disease patients have been performed, providing insight into the pathogenesis of those diseases, but reports regarding BS are limited. A microarray analysis comparing the gene expression of the peripheral blood from BS patients and healthy individuals revealed the upregulation of Th17 genes and interferon-related genes in BS [11]. Microarray analysis of CD4^+^ T cells and CD14^+^ monocytes from BS patients has also been reported, revealing the activation of the JAK/STAT pathway [12]. An RNA sequencing (RNA-seq) analysis of CD8^+^ T cells from BS patients has suggested an alteration of cAMP-mediated signaling in CD8^+^ T cells of BS patients [13]. However, analysis of the whole blood or only certain types of immune cells makes it difficult to identify cell types that are central to disease pathology and to analyze the interaction among the different cell types. To overcome those obstacles and to elucidate the role of each immune cell subset in the pathology of BS, we analyzed the data in ImmuNexUT [14], a comprehensive RNA-seq data of sorted immune cell subsets and eQTL database created by our group, with a focus on BS.

## Methods

### Patient and control group

Peripheral blood samples were obtained from 23 BS patients and 28 healthy donors. BS patients fulfilled the International Study Group criteria [15]. To detect the differences that are inherent in disease pathology, not the result of excessive inflammation, BS patients in remission or with low disease activity (the Behçet’s Disease Current Activity Form scores less than 3) were recruited [16]. As transcriptomic studies of the peripheral blood from patients with other autoimmune diseases have indicated that biologics induce profound changes in the transcriptome [17, 18], patients receiving biologics were excluded. The control group consisted of 28 healthy people, age- and gender-matched to the patient group. Written consent was obtained from the participants according to the Declaration of Helsinki principle, and the study was approved by the ethics committee of the University of Tokyo (G10095 [4]).

### Isolation and flow cytometric analysis (FCM) of the immune cells

Samples were collected as described previously [14]. Briefly, PBMCs were isolated from the peripheral blood by density gradient centrifugation with Ficoll-Paque Plus (GE Healthcare), and erythrocytes were lysed using ammonium chloride potassium buffer, as ammonium chloride potassium buffer has been reported to lyse erythrocytes efficiently without affecting the proportion of immune cells [19]. After blocking the non-specific binding of antibodies with Fc receptor binding inhibitor (eBioscience), the cells were stained with antibodies shown in Additional file 1The cells were analyzed and sorted using 8-color MoFlo XDP (Beckman Coulter) following the cell surface antigen-based definitions shown in Additional file 2. The results were analyzed using FlowJo version 10.5.0 (TreeStar Software). Neutrophils were isolated from EDTA anti-coagulated blood using MACSxpress Neutrophil Isolation Kit human and MACSxpress Erythrocyte Depletion Kit (Miltenyi Biotec).

### RNA-seq

PBMC subsets were collected into RLT (QIAGEN), and neutrophils were collected into TRIzol LS Reagent (Invitrogen). Total RNA was extracted using the RNeasy micro kit (QIAGEN). The SMART-seq v4 Ultra Low Input RNA Kit for Sequencing (Clontech) was used for cDNA library preparation, and sequencing was performed with HiSeq 2500 (Illumina) with a pair-end read of 100 base pairs. Sequencing data in BCL format was converted to FASTQ format using bcl2fastq2 v2.17.

### Quality control of RNA-seq data, mapping, and counting of reads

Adapter sequences were first removed from FASTQ format data using Cutadapt version 1.14. Next, using FASTX-Toolkit version 0.0.14, reads with Phred quality score below 20 were excluded. Mapping was performed using STAR version 2.5.3a with UCSC human genome 38 as the reference sequence [20]. Reads were counted using HTSeq version 0.9.1 [21]. Samples with uniquely mapped rates less than 80% or with uniquely mapped counts less than 5.00 × 10^6^ reads were removed from the analysis. The correlation coefficient between every pair of two samples from the same cell subset was calculated, and if the average of those correlation coefficients (Di) was less than 0.9, the sample was also removed from the analysis.

### Identification of differentially expressed genes (DEG)

Genes with a read count of less than 10 in 90% or more of the samples of the same subset were excluded, and normalization was performed using R package TCC version 1.22.0 [22]. After removing the batch effect using R package RUVseq version 3.24.0 [23], comparisons between the two groups were performed, using quasi-likelihood *F*-test in edge R version 1.16.0 [24]. Genes with a false discovery rate (FDR) less than 0.05 were considered significant.

### Weighted gene co-expression network analysis (WGCNA)

For weighted gene co-expression network analysis (WGCNA), genes with read counts less than 10 in 90% or more of the samples of the same subset were excluded. Count data was normalized with iterative DEGES/edgeR and converted to log 2 (CPM + 1) using R package TCC version 1.22.0. WGCNA was performed using WGCNA package version 1.64.1 [25] and iterative WGCNA version 1.1.6. VisNetwork was used to visualize the results (https://CRAN.R-project.org/package=visNetwork).

### eQTL analysis

The eQTL analysis was performed as described elsewhere [14]. After filtering out genes expressed at low levels in each cell subset (< 5 count in more than 80% samples or < 0.5 CPM in more than 80% samples), the expression data were normalized between samples with TMM, converted to CPM, and then normalized across samples using an inverse normal transform. A probabilistic estimation of expression residuals (PEER) method [26] was used to find hidden covariates. For each cell subset, a QTLtools permutation pass with 10,000 permutations was used to obtain gene-level nominal *p*-value thresholds corresponding to FDR < 0.05. A forward-backward stepwise regression eQTL analysis was subsequently preformed with a QTLtools conditional pass.

### Pathway analysis

ClusterProfiler was used to assess the enrichment of genes in modules with correlation with the diagnosis of BS to the KEGG pathways [27]. Pathways related to biological processes are depicted in the dotplot, and those related to diseases were omitted. Pathway analysis of modules with correlation to HLA-B51 was performed using Ingenuity Pathway Analysis 2.3 (QIAGEN Inc.).

### Tc17 score

Tc17 score was calculated using GSVA [28]. Genes included in the gene set are the following genes reported to be upregulated in Tc17 cells [29], especially pathogenic Tc17 cells [30]: *RORC*, *CCR6*, *IL23R*, *CD58*, *MAF*, *BLK*, *IL1R1*, *SOX13*, *SOCS3*, *CCL20*, *TCF7*, *RORA*, *NFKB1*, and *GZMB*.

### Statistical analysis

Categorical data were analyzed using Fisher’s exact test. Quantitative variables were compared using the Mann-Whitney *U* test. Correlation between two variables was evaluated by Pearson’s correlation coefficient or Spearman’s correlation coefficient. In multiple tests, Bonferroni’s method was used to correct *p*-values, and *p*-value < 0.05 was judged to be statistically significant. Statistical significance was separately described if other criteria were adopted. Graphs were generated using R version 3.5.1, R version 4.0.3, or GraphPad Prism version 9.0.0.

## Results

### Clinical characteristics of the participants

The clinical characteristics of participants are shown in Table 1. About half of the BS patients (47%) were HLA-B51-positive. At the time of participation in the study, 69% of the patients were taking colchicine, and 39% were taking steroids but the dosage was generally low. About half of the patients (56%) had at least one active symptom, most commonly skin lesions, arthralgia, or oral aphthous ulcers (Table 1). During the entire disease course, more than half of the patients had oral aphthous ulcerations, genital ulcers, uveitis, cutaneous lesions, and arthritis/arthralgia, but patients with intestinal lesions, vascular lesions, and neurological involvement were limited (Additional file 3).

**Table 1:** Clinical characteristics of BS patients and healthy controls

	BS patients (*n* = 23)	Healthy controls (*n* = 28)	
Female, *n* (%)	12 (52%)	21 (75%)	ns
Age, median (range)	54 (30–74)	56 (28–80)	ns
HLA-B51 positive, *n* (%)	11 (47%)		
Disease duration, years, median (range)	15 (1–37)		
Active symptoms at the time of participation in the study, n (%)	13 (56%)		
Oral aphthous ulcerations	3 (13%)		
Genital ulcers	0 (0%)		
Uveitis	0 (0%)		
Cutaneous lesions	7 (30%)		
Pathergy	2 (8%)		
Arthralgia/arthritis	5 (21%)		
Intestinal lesions	0 (0%)		
Vascular lesions	1 (4%)		
Neurologic disease	0 (0%)		
Treatment at the time of participation in the study, *n* (%, dosage)			
Colchicine	16 (69%, 0.25–1.5 mg/day)		
Prednisolone	9 (39%, 1.5–7.0 mg/day)		
Cyclosporine	5 (21%, 25–175 mg/day)		
Other immunomodulatory or immunosuppressive drugs (azathioprine, salazosulfapyridine, mesalazine, methotrexate)	7 (30%)		
No treatment	2 (8%)		

*ns* not significant

### Th17 cells are increased in the peripheral blood of BS patients

First, we compared the frequency of peripheral blood immune cells between HC and BS. The frequency of Th17 cells, defined as CD3^+^CD4^+^CD25^−^CD45RA^−^CXCR5^−^CCR6^+^CXCR3^−^ cells, was significantly increased in BS compared to HC, while no significant differences were found in CD8^+^ T cells, B cells, monocytes, or dendritic cells (Table 2, Additional file 4). We then compared the gene expression profile of immune cells between BS and HC (Additional files 5 and 6). DEG in many subsets included many immunologically important genes, including *CXCL8*, *RGS1*, *STAT6*, and cytokine receptors (Additional file 7). For Th17 cells, we identified 58 DEG, and those genes made a clear distinction between BS and HC (Additional file 8). Pathway analysis of the DEG suggested activation of the NFκB pathway in Th17 cells in BS (Additional file 9).

**Table 2:** Cell subset frequencies in BS patients and in healthy controls

Parent population	Subset	BS patients (*n* = 23)	Healthy controls (*n* = 28)	*p*-value
Lymphocyte	CD4^+^ T cells	36.5 ± 10.8%	37.4 ± 7.0%	0.80
	Naïve CD4^+^ T cells	16.8 ± 8.6%	17.7 ± 6.7%	0.55
	Memory CD4^+^ T cells	16.3 ± 7.3%	15.8 ± 5.0%	0.90
	CD8^+^ T cells	16.7% ± 7.0%	14.0 ± 5.0%	0.13
	Naïve CD8^+^ T cells	9.1 ± 5.4%	8.4 ± 4.7%	0.86
	Memory CD8^+^ T cells	7.6 ± 4.4%	5.7 ± 2.3%	0.19
	B cells	11.3 ± 6.7%	10.8 ± 3.8%	0.94
	Natural killer cells	12.5 ± 10.8%	11.9 ± 6.5%	0.45
B cells	Naïve B cells	69.6 ± 21.5%	73.2 ± 8.6%	0.60
	Unswitched memory B cells	7.9 ± 8.1%	7.3 ± 3.5%	0.19
	Switched memory B cells	16.0 ± 12.4%	14.3 ± 5.4%	0.65
	Plasmablasts	1.7 ± 2.6%	1.1 ± 1.0%	0.86
	Double negative B cells	3.7 ± 2.6%	3.4 ± 1.6%	0.79
CD4+ T cells	Th1 cells	9.2 ± 5.1%	13.1 ± 5.4%	0.003
	Th2 cells	11.7 ± 5.5%	9.7 ± 4.6%	0.11
	Th17 cells	9.5 ± 4.9%	5.4 ± 2.4%	< 0.0001
	T follicular helper cells	4.9 ± 3.1%	6.5 ± 3.4%	0.11
	Fraction II effector regulatory T cells	1.4 ± 0.5%	1.3 ± 0.4%	0.34
Lymphocyte and monocyte gate	Monocytes	18.6 ± 9.9%	13.4 ± 6.5%	0.06
	CD16^+^ monocytes	1.6 ± 1.1%	1.4 ± 0.9%	0.66
	CD16^−^ monocytes	17.0 ± 9.1%	11.9 ± 6.1%	0.06
	Dendritic cells	0.7 ± 0.3%	0.7 ± 0.3%	0.60
	Myeloid dendritic cells	0.5 ± 0.2%	0.5 ± 0.2%	0.34
	Plasmacytoid dendritic cells	0.2 ± 0.1%	0.2 ± 0.1%	0.63

Nominal *p*-values from Mann-Whitney’s *U* test are indicated

### Gene expression profiles of various immune cells show correlation with clinical features of BS

**Fig. 1:**
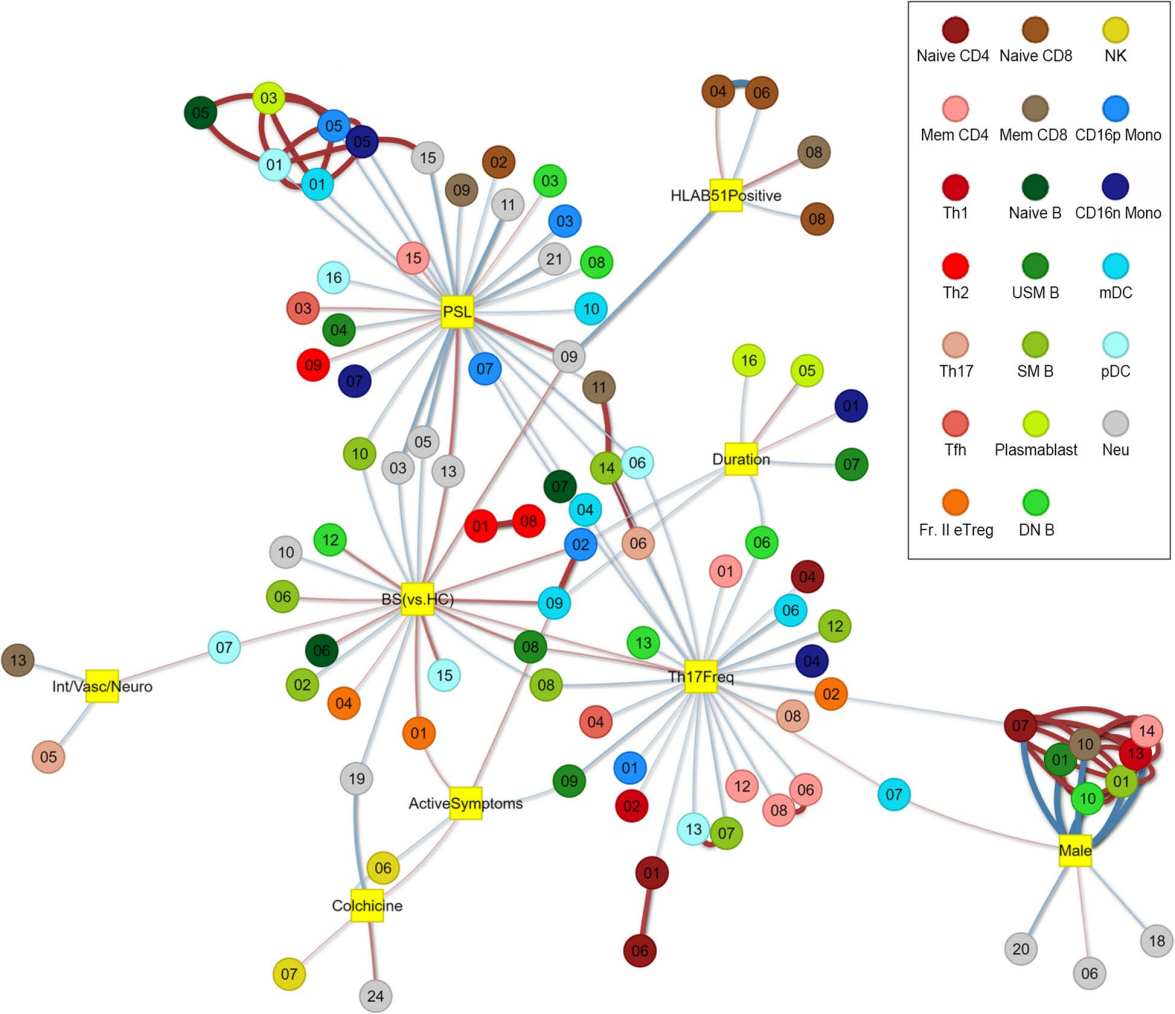
Gene expression profiles of various immune cells show a correlation with clinical features of BS. Gene modules consisting of genes with similar co-expression patterns in each cell subset were identified using WGCNA. Each circle represents a module with the colors indicating the cell subset of the module and the numbers indicating the module number. Modules with significant correlation with clinical parameters are visualized. Blue lines represent the negative correlation, and red lines represent the positive correlation with line widths indicating the absolute value of correlation coefficients. The squares indicated clinical parameters. Int/Vasc/Neuro, BS patients with intestinal, vascular, and neurological involvement compared to BS patients without those organs involved. Duration: disease duration

Next, we sought to investigate the association of gene expression profiles in various immune cell subsets with clinical parameters. We first performed WGCNA to identify gene modules consisting of genes with similar co-expression patterns. We then assessed the correlation of those modules with clinical parameters. A total of 247 modules were identified in 20 subsets (Additional files 10 and 11). Modules from various immune cells, including CD4^+^ T cells, CD8^+^ T cells, B cells, NK cells, monocytes, dendritic cells (DCs), and neutrophils, showed significant correlation with either the diagnosis of BS or clinical features of BS patients, suggesting the contribution of various immune cells to the pathogenesis of BS (Fig. 1), and some of those modules were further examined.

### Pathways of inflammatory chemokines and cytokines are activated in antigen-presenting cells of BS patients

**Fig. 2:**
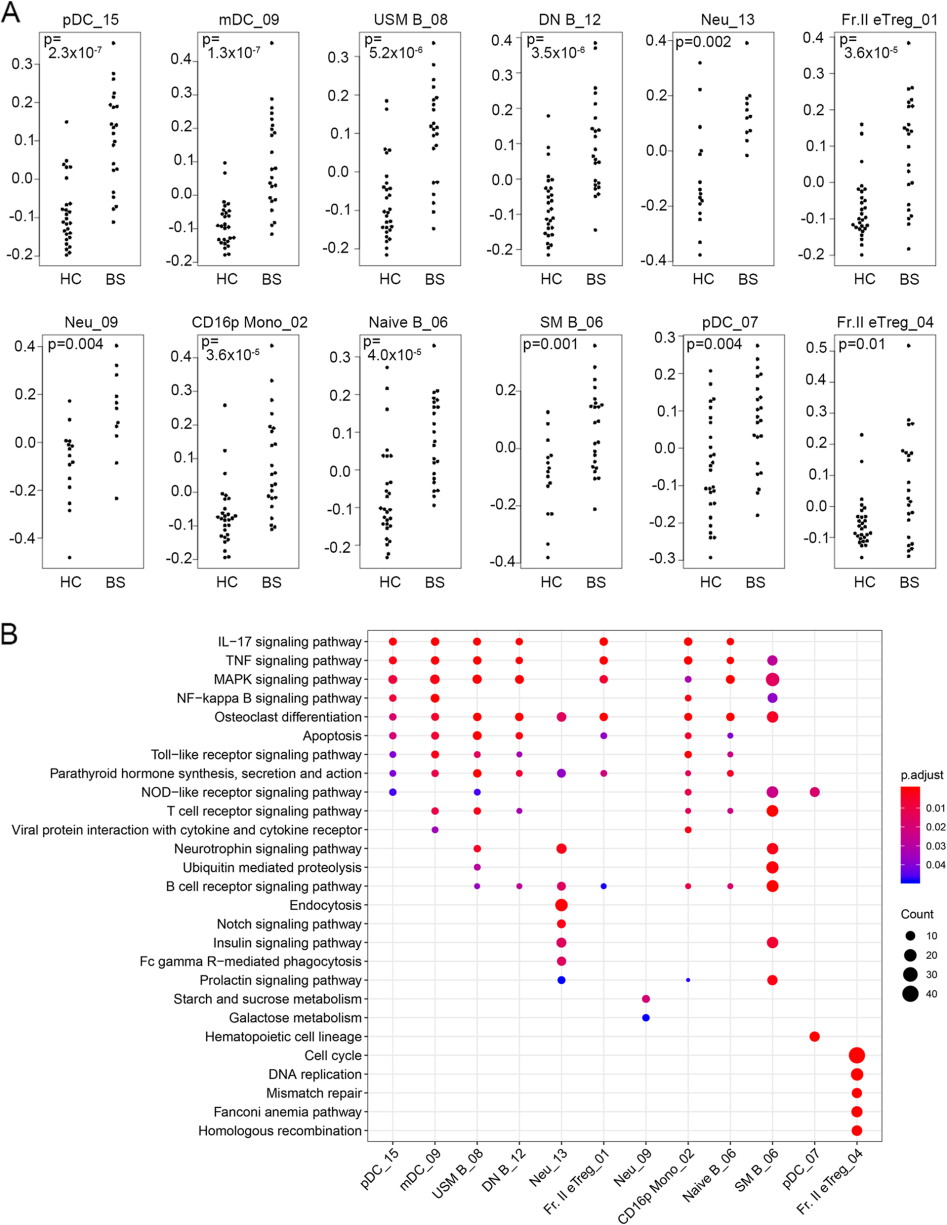
Pathways of inflammatory chemokines and cytokines are activated in antigen-presenting cells of BS patients. **A** Eigengenes of modules with a positive correlation of BS in healthy controls and BS patients. **B** Pathway analysis of modules with positive correlation with the diagnosis of BS

To further examine the relationship between gene modules and clinical parameters, we first focused on modules that were upregulated in BS patients compared to HC. Modules in antigen-presenting cells such as myeloid DC, plasmacytoid DC (pDC), CD16^+^ monocyte, and B cells (unswitched memory B cells, double-negative B cells, naïve B cells, switched memory B cells) showed a significant correlation with the diagnosis of BS, and eigengenes of these modules were higher in BS patients compared to healthy controls (Fig. 2A). Pathway enrichment analysis of those modules suggested that inflammatory cytokines, MAPK pathways, and NFkB pathways were upregulated in these subsets (Fig. 2B). These results suggest the activation of pathways related to chemokines and inflammatory cytokines and pathways mediated by MAPK and NFκB in antigen presenting cells of BS patients.

### Genetic risk factors may account for the differences in the expression of BS-associated modules

To elucidate the relationship between genetic risk factors and the changes in the transcriptome that we observed in BS patients, we examined the eQTL catalog in ImmuNexUT [14]. The *IL10* locus has been associated with BS, and it has been reported that the BS risk allele at rs1518111 decreases the production of *IL10* in monocytes and macrophages [5, 31]. Our eQTL analysis was consistent with these results (Additional file 12). Furthermore, rs4683184, a BS risk SNP located in the *CCR1*-*CCR3* locus, had an eQTL effect on multiple chemokine receptors (Additional file 13).

**Fig. 3:**
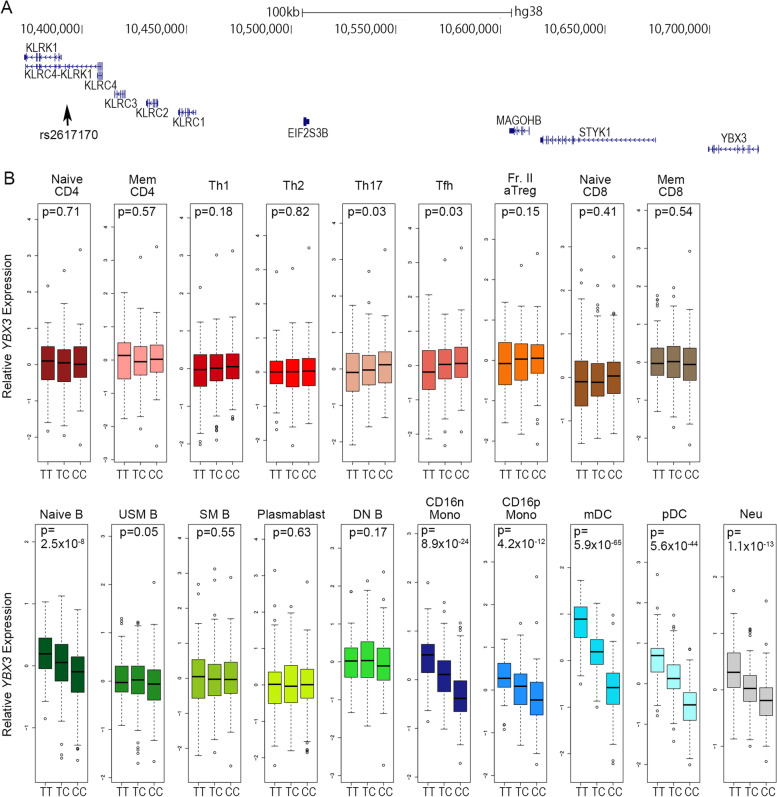
BS risk SNP rs2617170 modulates the expression of *YBX3*. **A** Relationship of rs2617170 with nearby genes. **B** eQTL effect of rs2617170 on *YBX3*. Residuals after normalization are plotted by genotype

In addition, rs2617170, which has been reported to be associated with BS [6, 10], is a missense SNP in *KLRC4*, and *KLRC4* has been reported as the responsible gene at this locus (Fig. 3A). However, this SNP has pleotropic effects and influences the expression of various genes, including *KLRC4*, *KLRC1*, *KLRK1*, and *YBX3* (Fig. 3B, Additional file 14). *YBX3*, whose expression was increased in individuals with the risk allele, is a member of the pDC_15 module (Additional file 15) associated with the diagnosis of BS. Thus, the genetic risk for BS increases the expression of *YBX3*; therefore, in addition to the changes in the KLRC4 protein induced by rs2617170, rs2617160 may contribute to disease pathogenesis by altering the expression pattern of *YBX3*.

### A Memory CD8^+^ T cell module with IL-17-associated genes shows correlation with HLA-B51 positivity

**Fig. 4:**
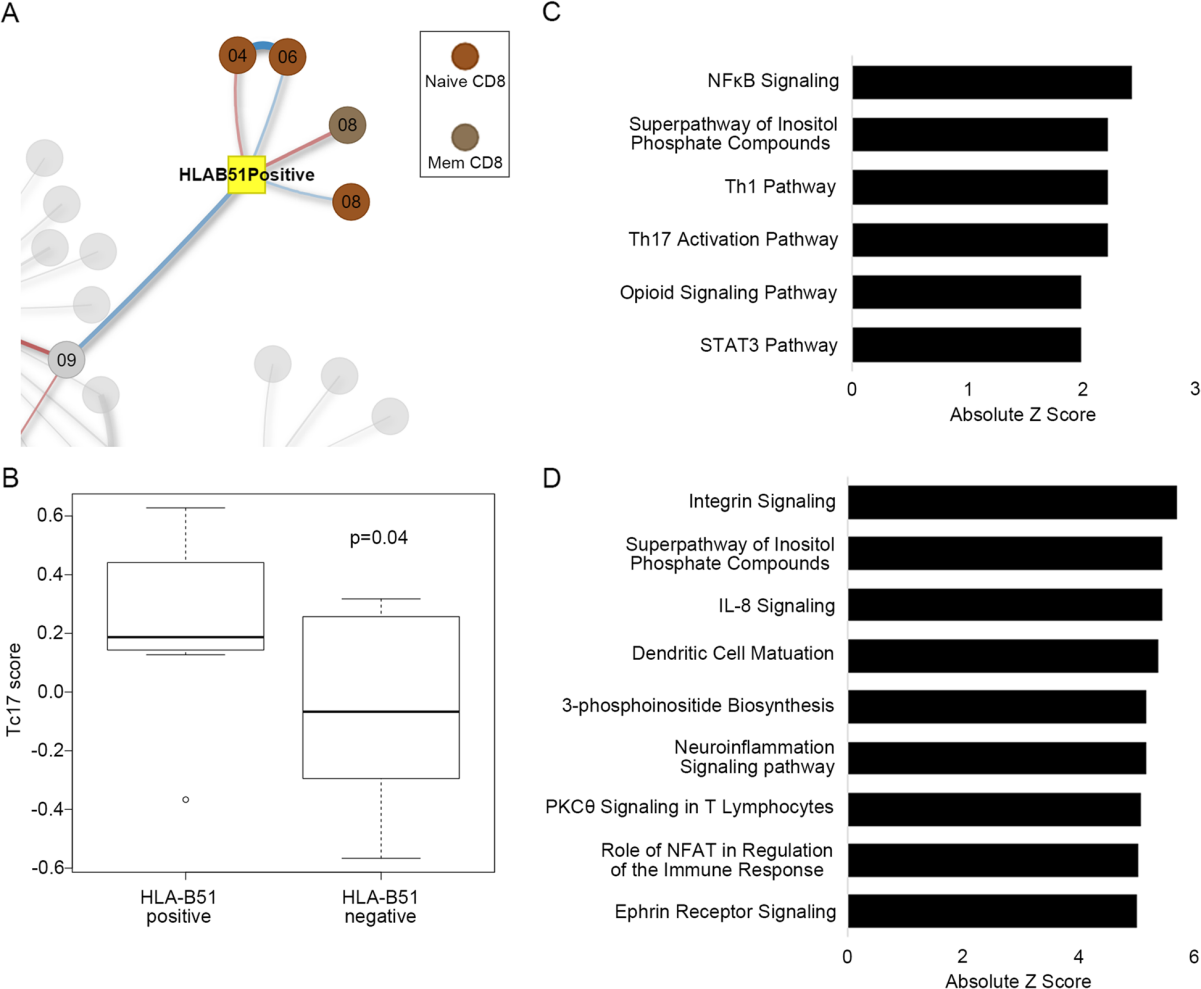
A memory CD8+ T cell module with IL-17-associated genes shows a correlation with HLA-B51 positivity. **A** An enlarged view of modules showing correlation with HLA-B51 positivity in BS patients. **B** Tc17 score in HLA-B51-positive patients (*n* = 7) compared to HLA-B51-negative patients (*n* = 17). **C** Pathway analysis of “MCD8_08,” the module with the strongest positive correlation with HLA-B51 positivity. Pathways with absolute *z* scores ≧ 2 are shown. **D** Pathway analysis of “NCD8_06,” the module with the strongest negative correlation with HLA-B51 positivity. Pathways with absolute *z* scores ≧ 5 are shown

HLA-B51 positivity is a strong risk factor for BS, but the role of HLA-B51 in the pathogenesis of BS has not been elucidated. To investigate this point, we focused on modules that showed a correlation with HLA-B51 positivity in BS. A module from memory CD8^+^ T cells “MCD8_08” showed the greatest correlation with HLA-B51 positivity in BS patients (Fig. 4A). This module included genes associated with IL-17 producing CD8+ T cells, Tc17 cells, for example, *RORC*, *IL23R*, and *CCR6* [32] (Additional file 14), and Tc17-associated gene expression score (Tc17 score) was higher in HLA-B51-positive patients compared to HLA-B51-negative patients (Fig. 4B). Pathway analysis for the module “MCD8_08” suggested activation of NF-κB signaling, Th17 activation pathway, Th1 pathway, and STAT3 pathway (Fig. 4C).

In contrast to modules from memory CD8^+^ T cells showing a positive correlation with HLA-B51 positivity in BS patients, modules of naïve CD8^+^ T cells showed a negative correlation with HLA-B51 positivity in BS. Pathway analysis of the module “NCD8_06” showed possible activation of pathways of integrins and chemokines in naïve CD8^+^ T cells in HLA-B51-negative BS patients (Fig. 4D). These results suggest that CD8^+^ T cells may play a different role in HLA-B51-positive BS and HLA-B51-negative BS.

## Discussion

We herein reported our FCM and transcriptome analysis of immune cells of BS patients. FCM analysis revealed an increase in Th17 cells in the peripheral blood of BS patients. Various reports have implicated a role for IL-17 and Th17 cells in BS [33–35], and our findings are consistent with those reports. Our analysis of the transcriptome of Th17 cells from BS patients suggested the activation of the NFκB pathway in Th17 cells of BS patients. NFκB transcription factor c-Rel has been reported to be essential for the differentiation of Th17 cells [36], and the activation of the NFκB pathway seen in Th17 cells of BS patients may contribute to the increased number of Th17 cells in BS patients. An increase in Th17 cells has also been reported in other autoimmune diseases, including early systemic sclerosis [37], suggesting that the role of Th17 cells in autoimmunity may be a common theme in many autoimmune diseases, not just BS.

In addition, transcriptome analysis of peripheral blood immune cell subsets using WGCNA indicated that an increase in the expression of genes related to IL-17 production in CD8^+^ memory cells of HLA-B51-positive BS patients, and this may reflect an increase in the number or function of Tc17 cells, IL-17-producing CD8^+^ cells. Previous reports [33–35] regarding the role of IL-17 in BS have mainly focused on CD4^+^ IL-17-producing cells, Th17 cells; however, it has been reported that a significant proportion of IL-17-producing cells in BS patients are CD4-negative [33], and our results suggest that these cells may be Tc17 cells. Despite being well known as a strong genetic risk factor for BS, the role of HLA-B51 in the pathogenesis of BS has remained elusive. Some have suggested that aberrant activation of CD8^+^ T cells due to the HLA-B51 contributes to the pathogenesis of BS [38], while others emphasize the role of interactions with other immune cells, such as NK cells, and HLA-B51 [39]. Our data suggests the importance of CD8^+^ T cells in the disease pathology of HLA-B51 patients. That is, in HLA-B51-positive patients, genetic factors may promote the differentiation of Tc17 cells, and these cells may serve as a potential therapeutic target in BS.

Another important finding from the transcriptomic analysis in this study is the association of gene expression profiles in antigen-presenting cells, such as myeloid DC, pDC, CD16-positive monocyte, and B cells, with BS. Pathway analysis of those modules indicated that pathways related to inflammatory cytokines, as well as the MAPK pathway and NFκB pathway, are upregulated in those cell subsets in BS. It has been reported that activated macrophages play an important role in the differentiation of human Th17 cells [40], and the activation of DCs may promote the differentiation of Th17 cells and contribute to the increase in Th17 cells in BS patients.

Analysis of transcriptomic data together with an eQTL database, ImmuNexUT, allowed us to identify possible links between the gene expression and genetic risk factors for BS. For example, rs2617170 is a missense SNP in *KLRC4*, and in addition to altering the amino acid sequence, the eQTL catalog in ImmuNexUT indicated that it also acts as an eQTL for *KLRC4*, consistent with a previous report by Yang et al. [10]. Furthermore, we observed eQTL effects on multiple genes in multiple cell types, including the expression of *YBX3* in pDCs. The pleotropic effect of rs2617170 may be due to it acting as an enhancer for these genes, in addition to being a missense SNP. As *KLRC4* influences the function of NK cells, the interaction between NK cells and DCs may also account for the eQTL effect on *YBX3* in DCs [41]. *YBX3* is included in a pDC module associated with BS, and genetic risk factors may cause changes in the expression pattern of *YBX3* in BS patients. YBX3, encoded by *YBX3*, is an RNA-binding protein that has been reported to bind to various genes, regulating their expression, and one of the genes reported to be regulated by YBX3 is *JAK1* [42], which has been implicated in the pathogenesis of BS from both genetic studies [43] and from clinical studies [12]. In addition, YBX3 controls amino acid levels by influencing mRNA abundance of SLC7A5 and SLC3A2 [42], and as immunometabolism has been implicated in the function of various immune cells including DCs [44], YBX3 may contribute to the pathogenesis of BS by altering the function of DCs and is a potential therapeutic target.

There are several limitations to this study. First, the number of patients was small, and 92% of the patients were receiving therapy for BS, making it difficult to discriminate the effect of treatment and the effect of the disease itself in certain instances. In addition, patients with high disease activity were excluded, and different changes may be seen in patients with highly active disease.

## Conclusions

FCM analysis and transcriptome analysis of BS patients revealed an increase in Th17 cells in the peripheral blood and activation of the NFκB pathway in those cells. iWGCNA analysis revealed the association of BS with genes related to antigen-presenting cell activation, and *YBX3*, whose expression in pDC is influenced by a BS risk polymorphism, rs2617170, was identified as a potential key molecule, connecting genetic risk factors of BS with activation of antigen-presenting cells in BS. Furthermore, HLA-B51, an important genetic risk factor of BS, was associated with IL-17-associated genes in memory CD8^+^ T cells, suggesting a role for Tc17 cells in the pathogenesis of BS. In conclusion, our analysis suggested that Tc17 cells and *YBX3* as potential therapeutic targets in BS.

## Supplementary Information

**Additional file 1.** Antibodies used for flow cytometry.

**Additional file 2.** Definition of PBMC subsets.

**Additional file 3.** Clinical symptoms of the BS patients during the entire disease course.

**Additional file 4.** Th17 cells are increased in the peripheral blood of BS patients.

**Additional file 5.** Genes upregulated in BS patients in each cell subset.

**Additional file 6.** Genes downregulated in BS patients in each cell subset.

**Additional file 7.** MA plot of each cell subset.

**Additional file 8.** Heatmap of DEG in Th17 cells.

**Additional file 9.** Pathway Analysis of DEG between BS Patients.

**Additional file 10.** Top 20 members of modules with significant correlation with clinical parameters.

**Additional file 11.** Modules and their relationship to clinical parameters.

**Additional file 12 **eQTL effect of rs1518111 on *IL10*.

**Additional file 13 **eQTL effect of rs4683184 on *CCR1*, *CCR2*, *CCR3*, and *CCR5*.

**Additional file 14 **eQTL effect of rs2617170 on *KLRK1*, *KLRC4*, and *KLRC1*.

**Additional file 15.** Members of “pDC_15” associated with the diagnosis of BS.

**Additional file 16.** Members of “MCD8_08”, the module with strongest positive correlation with HLA-B51 positivity.

## Data Availability

The RNA-seq data analyzed during the current study and the eQTL catalog are available at the National Bioscience Database Center (NBDC) (E-GEAD-397, E-GEAD-398, and E-GEAD-420) (https://biosciencedbc.jp/en/).

## Abbreviations

BS: Behçet’s syndrome
WGCNA: Weighted gene co-expression network analysis
pDC: Plasmacytoid dendritic cell
TNF-α: Tumor necrosis factor-α
FCM: Flow cytometric analysis
RNA-seq: RNA sequencing
DEG: Differentially expressed genes
PEER: Probabilistic estimation of expression residuals
HC: Healthy controls
