# Supplementary material for: Transcriptome analysis of immune cells from Behçet’s syndrome patients: the importance of IL-17-producing cells and antigen-presenting cells in the pathogenesis of Behçet’s syndrome
Source: Arthritis Res Ther. 2022 Aug 8;24:186. doi: 10.1186/s13075-022-02867-x (PMC9358821; doi:10.1186/s13075-022-02867-x)
Supplement: Supplementary file 1 — Additional file 1. Antibodies used for flow cytometry. [file 13075_2022_2867_MOESM1_ESM.pdf]

Additional file 1. Antibodies used for flow cytometry

Sorting panel group	Antibodies (clone, company)
$CD4^{+}$ T cells	Alexa Fluor 488 anti-CD25 (clone BC96, eBioscience)<br>PerCP-Cy5.5 anti-CXCR5 (clone RF8B2, BD)<br>PE-Cy7 anti-CD3 (clone UCHT1, BioLegend)<br>APC anti-CCR6 (clone 11A9, BD)<br>APC-Cy7 anti-CD45RA (clone HI100, BioLegend)<br>Brilliant Violet 421 anti-CXCR3 (clone 1C6/CXCR3, BD)<br>V500 anti-CD4 (clone RPA-T4, BioLegend)
B cells and CD8+ T cells	FITC anti-CD27 (clone O323, BioLegend)<br>PE anti-CD19 (clone HIB19, BioLegend)<br>PerCP-Cy5.5 anti-CD38 (HIT2, BioLegend)<br>PE-Cy7 anti-CD3 (clone UCHT1, BioLegend)<br>APC anti-CD45RA (clone HI100, BioLegend)<br>APC-Cy7 anti-CD4 (clone RPA-T4, BioLegend)<br>Brilliant Violet 421 anti-IgD (clone IA6-2, BD)<br>V500 anti-CD8a (clone RPA-T8, BD)
Natural killer cells, monocytes, and dendritic cells	FITC anti-CD14 (clone M5E2, BioLegend)<br>PE anti-HLA-DR (clone L243, eBioscience)<br>PerCP-Cy5.5 anti-CD16 (clone 3G8, BioLegend)<br>PE-Cy7 anti-CD3 (clone UCHT1, BioLegend)<br>APC anti-CD123 (clone AC145, Miltenyi)<br>APC-Cy7 anti-CD56 (clone HCD56, BioLegend)<br>Brilliant Violet 421 anti-CD11c (clone B-ly6, BD)<br>V500 anti-CD19 (clone HIB19, BD)
