# Supplementary material for: Transcriptome analysis of immune cells from Behçet’s syndrome patients: the importance of IL-17-producing cells and antigen-presenting cells in the pathogenesis of Behçet’s syndrome
Source: Arthritis Res Ther. 2022 Aug 8;24:186. doi: 10.1186/s13075-022-02867-x (PMC9358821; doi:10.1186/s13075-022-02867-x)
Supplement: Supplementary file 2 — Additional file 2. Definition of PBMC subsets. [file 13075_2022_2867_MOESM2_ESM.pdf]

Additional file 2. Definition of PBMC subsets

Subset name (abbreviation)	Definition according to cell surface markers
CD4$^+$ T cells	CD3$^+$CD4$^+$
Naïve CD4$^+$ T cells (Naïve CD4)	CD3+CD4+/CD25-/CD45RA+
Memory CD4$^+$ T cells (Mem CD4)	CD3+CD19-/CD4-CD8+/CD45RA-
Th1 cells (Th1)	CD3+CD4+/CD25-/CD45RA-CXCR5-/CCR6-CXCR3+
Th2 cells (Th2)	CD3+CD4+/CD25-/CD45RA-CXCR5-/CCR6-CXCR3-
Th17 cells (Th17)	CD3+CD4+/CD25-/CD45RA-CXCR5-/CCR6+CXCR3-
T follicular helper cells (Tfh)	CD3+CD4+/CD25-/CD45RA-CXCR5+
Fraction II effector regulatory T cells (Fr. II eTreg)	CD3+CD4+/CD25++LAG3-/CD45RA-
CD8$^+$ T cells	CD3+CD19-/CD4-CD8+
Naïve CD8$^+$ T cells (Naïve CD8)	CD3+CD19-/CD4-CD8+/CD45RA+
Memory CD8$^+$ T cells (Mem CD8)	CD3$^+$CD19$^-$CD4$^-$CD8$^+$CD45RA$^-$
B cells	CD3-CD19+
Naïve B cells (Naïve B)	CD3-CD19+/IgD+CD27-
Unswitched Memory B cells (USM B)	CD3-CD19+/IgD+CD27+
Switched Memory B cells (SM B)	CD3-CD19+/IgD-CD27+
Plasmablasts (Plasmablast)	CD3-CD19+/IgD-/CD27++CD38+
Double negative B cells (DN B)	CD3-CD19+/IgD-CD27-
Natural killer cells (NK)	CD3-CD19-/CD14-/CD56+
Monocytes	CD3$^-$CD19$^-$HLA-DR$^+$CD56$^-$CD14$^+$
CD16$^+$ monocytes (CD16pMo)	CD3-CD19-/HLADR+/CD56-/CD14+CD16+
CD16$^-$ monocytes (CD16nMo)	CD3-CD19-/HLADR+/CD56-/CD14+CD16-
Dendritic cells	CD3-CD19-/CD56-CD16-/HLADR+CD14-
Myeloid dendritic cells (mDC)	CD3-CD19-/CD56-CD16-/HLADR+CD14-/CD11c+CD123-
Plasmacytoid dendritic cells (pDC)	CD3-CD19-/CD56-CD16-/HLADR+CD14-/CD11c-CD123+
