# Supplementary material for: Transcriptome analysis of immune cells from Behçet’s syndrome patients: the importance of IL-17-producing cells and antigen-presenting cells in the pathogenesis of Behçet’s syndrome
Source: Arthritis Res Ther. 2022 Aug 8;24:186. doi: 10.1186/s13075-022-02867-x (PMC9358821; doi:10.1186/s13075-022-02867-x)
Supplement: Supplementary file 3 — Additional file 3. Clinical symptoms of the BS patients during the entire disease course. [file 13075_2022_2867_MOESM3_ESM.pdf]

**Additional file 3. Clinical symptoms of the BS patients during the entire disease course**

| Symptom, n (%) | Presence of the symptom during the disease course (n=23) |
|---|---|
| Oral aphthous ulcerations | 23 (100 %) |
| Genital ulcers | 17 (73 %) |
| Uveitis | 12 (52 %) |
| Cutaneous lesions | 23 (100 %) |
| Pathergy | 7 (30 %) |
| Arthralgia / Arthritis | 13 (56 %) |
| Intestinal lesions | 5 (21 %) |
| Vascular lesions | 5 (21 %) |
| Neurologic disease | 1 (4 %) |
