# Supplementary material for: Transcriptome analysis of immune cells from Behçet’s syndrome patients: the importance of IL-17-producing cells and antigen-presenting cells in the pathogenesis of Behçet’s syndrome
Source: Arthritis Res Ther. 2022 Aug 8;24:186. doi: 10.1186/s13075-022-02867-x (PMC9358821; doi:10.1186/s13075-022-02867-x)
Supplement: Supplementary file 4 — Additional file 4. Th17 cells are increased in the peripheral blood of BS patients. [file 13075_2022_2867_MOESM4_ESM.pdf]

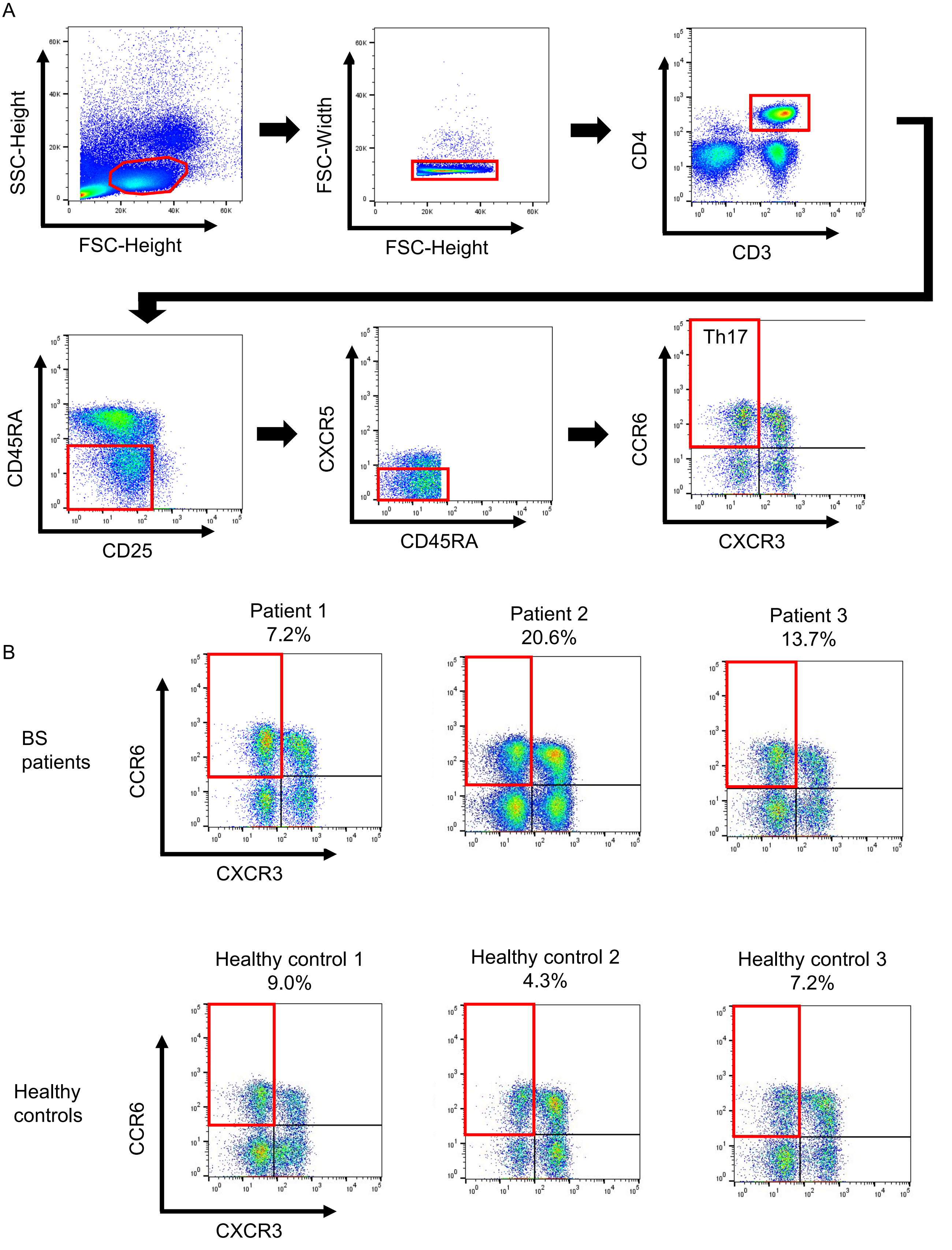

Additional file 4. Th17 cells are increased in the peripheral blood of BS patients

（A） Gating strategy used for Th17 cells

（B） FACS plots of selected BS patients and healthy controls. The numbers indicated the percentage of Th17 cells ($CD3^+CD4^+CD25^-CD45RA^-CXCR5^-CCR6^+CXCR3^-$ cells) among $CD3^+$ $CD4^+$ T cells.
