# Supplementary material for: Transcriptome analysis of immune cells from Behçet’s syndrome patients: the importance of IL-17-producing cells and antigen-presenting cells in the pathogenesis of Behçet’s syndrome
Source: Arthritis Res Ther. 2022 Aug 8;24:186. doi: 10.1186/s13075-022-02867-x (PMC9358821; doi:10.1186/s13075-022-02867-x)
Supplement: Supplementary file 5 — Additional file 5. Genes upregulated in BS patients in each cell subset. [file 13075_2022_2867_MOESM5_ESM.pdf]

Additional file 5. Genes upregulated in BS patients in each cell subset

Top 30 genes (in the order of p values) upregulated in each cell subset. For subsets with less than 30 genes with FDR<0.05, only genes with FDR<0.05 are shown.

Naive CD4	Mem CD4	Th1	Th2	Th17	Tfh	Fr II eTreg
CD300E	LILRA1	COPA	TLR8	TLR7	RAD54L2	FOS
ANPEP	HK3	PKP2	SIGLEC5	RCSD1	LCP1	PTGS2
LILRA6	TNFSF8	HK1	NFX1	TMEM30A	DUSP1	DUSP1
PDGFRB	UACA	LCP1	RCSD1	IARS2	TRAF5	CD69
CHST11	TMEM176B	USP4	GPR15	BRAP	RCSD1	RGS1
RCSD1	CD19	MADD	CAPN2	KCTD20	LOC101928994	JUNB
LILRA1	CD300E	PRPF8	STAB1	FKBP5	FOS	HK1
CAPN2	FCRLA	ESYT2	PLCL2	ZDHHC20	HK1	DUSP2
FOS	CSF3R	ESYT1	ODF2	MBTPS1	ZKSCAN1	DNAJB1
PSMD9	CHST11	OSBP	GCFC2	NDN	ANKRD13A	MCM4
FCN1	FCN1	CANX	ZC3H11A	UBC	MMP17	AK2
LILRA2	FES	RASSF5	FKBP5	UBE3C	GPR15	CXCL8
PIK3R5	MPEG1	ARHGEF6	ANPEP	COPA	CAPN2	PMAIP1
	KCTD20	NBR1	MBTPS1	NFX1	ITGAL	NR4A2
	GPR15	GNS	TLR4	FOS	FKBP5	RGS2
	TBC1D9	DUSP1	VCAN	NAT10	ESYT2	JUN
	MS4A7	MBTPS1	TTC17	CYFIP2	ZBTB16	GNB1
	CLEC7A	API5	CNOT6	ESYT2	COPA	LINC00200
	MEF2C	NMT1	TNFSF8	IL2RA	PITPNA	CHN2
	IL2RA	EPGN	SLC38A1	KIAA0100	IARS2	SLMAP
	CD1D	SNORA80B	TRIM37	LCP1	MCM4	MBTPS1
	IARS	GGA2	ESYT2	CKAP5	AMMECR1L	FOSB
	SYK	DIS3L	PARP4	IKBKAP	JUN	CRISPLD2
	ADSL	NUMB	RPS6KA3	EXOC2	UBC	LCP1
	GAPT	FKBP5	ZNF430	NUP50	NBPF12	PTPN7
	KCNJ1	EDEM1	NUMB	FBXO18	LETM1	ALDH18A1
	ZKSCAN1	PARP4	TMEM176B	RASSF5	PSMD2	PPP1R15A
	BTK	ANXA2	DUSP1	ZC3H12C	DOCK7	PLK4
	ACAD10	NLRC3	GJB6	ITGAL	CASK	NCAPG2

Naive CD8	Mem CD8	Naive B	USM B	SM B	Plasmablast	DN B
None	HK1	MYO10	DUSP1	KIAA0100	FCGR3B	RGS2
	FOSB	UBC	UBC	MTTP	KLF4	RGS1
	DUSP1	DUSP1	RGS1	PHC1	RGS1	DUSP1
	YLPM1	PTPRB	NR4A2	ZNF160	CD93	CXCL8
	FKBP5	ZFAT	CD69	DENND4C	CRISPLD2	FOS
	DGCR9	RGS2	RGS2	DIS3L	ACTN1	CD69
	LOC101926892	RGS1	FOS	YME1L1	P2RY13	NR4A2
	SVIL	SMPDL3A	KLF6	SNX19	FOS	CENPU
	CFH	ANPEP	FCRLA	TAB3	TNFRSF10C	PMAIP1
	PLEKHG3	DPP4	JUN	GUCY1B3	MICAL2	UBC
	POLH	JUN	PPP1R15A	INPP5D	CXCL8	JUNB
	CHST11	PPP1R15A	GLUD1	ANKRD13A	RTN1	PPP1R15A
	LIMK2	KLF6	MBTPS1	SNRNP200	DYSF	RBM3
	EDN3	C1orf186	WARS	MTMR12	MAFB	H3F3B
	FOS	PAPSS2	HSPA5	NCAPD3	MPEG1	CTAG2
	KDM4A	RBM3	MOAP1	STAT6	AQP9	DUSP2
	NR4A2	COX11	KCNIP2	PLCG2	GIMAP4	DTD1
	ESYT2	WARS	ARRDC3	ATG13	DUSP1	SBDSP1
	PTPN7	TECTA	DDIT4	STAT3	CD69	HEXB
	MYC	FAF1	TLE1	RABGAP1L	CD5	FOSB
		NFX1	FOSB	NFX1	CXCR2	KLF6
		NLRC5	IRAK1	NFATC2	LPCAT2	NFKBIA
		CD44	RBM3	VPS39	RGS2	EIF1
			LOC101929162	CEP250	LILRA6	JUN
			TPM3	NBPF12	PADI4	TMED3
			YBX3	ZFP1	SECTM1	TSC22D3
			LOC285074	LOC101929162	LYZ	IL1B
			RFX5	PARP4	PLXDC2	NFKBIZ
			ZNF792	RASSF2	EPB41L3	MAATS1

NK	CD16pMo	CD16nMo	mDC	pDC	Neu
VCAN	RGS1	DDIT4	UBC	FOS	TSHZ3
LGALS2	DUSP1	RGS1	CXCL8	RGS2	RIN3
TNFRSF19	FOSB	NR4A2	DUSP1	DUSP1	ARRB2
PDCD1	CXCL8	ARL4A	RGS1	JUNB	SEMA4D
	DDIT4	CXCL8	PPP1R15A	RGS1	ABTB1
	CCL3L1	PIK3AP1	FOS	NR4A2	OS9
	NR4A2	TEX2	DDIT4	PLK2	ZYX
	UBC	GCNT1	CTAG2	STAT6	STXBP2
	G0S2	ATP6V0A2	PMAIP1	DDIT4	ARL8A
	GPER1	HCAR3	ARL4A	CXCL8	ABR
	NBR1	DOCK2	CXCR4	PMAIP1	GDI1
	FOS	ID2	JUN	JUN	PXN
	FLT3	ACLY	DUSP2	ZNF146	FCHSD1
	ZDHHC3	UBC	HSPA5	NR4A3	ELOVL1
	LCP1	POLG	RGS2	NISCH	RASGRP4
	EPB41L3	LCP1	FOSB	LCP1	MAPK3
	DHX32	G0S2	SRGAP1	AGO4	PLEKHM1
	FKBP5	ADAMTS2	YRDC	FOSB	PDLIM7
	XRCC6	AREG	OTUD1	NAIP	LRP10
	NCOA4	FAM65B	KLF4	KCTD20	CFL1
	BRE-AS1	RIC1	CD69	DOCK2	GPSM3
	RIC1	STAT6	IER2	DUSP6	KCTD2
	HADHA	PARP4	DNTTIP2	PARP4	CBX4
	MAPKAP1	BTK	CSRNP1	KLF4	MTMR3
	DDX19B	MAPKAP1	CCL3L1	SOCS3	GNAS
	SND1	HCAR2	PLEK	PARP6	VPS37B
	JUN	FNIP2	KIAA0319L	WARS	TFEB
	MBTPS1	COPA	AREG	KIAA0196	PREX1
	SF3B3	DUSP2	FNIP2	ARL4A	DNM2

Naive CD8	Mem CD8	Naive B	USM B	SM B	Plasmablast	DN B
None	HIST1H4C	CLK1	CLK1	HIST1H4C	INSIG2	CLK1
	CLK1	MED21	RPL32P3	SNORD13	FAM200A	ID3
	DPM1	HBP1	NOP10	NOP10	HERPUD1	P2RY10
	PIGF	CLK4	PCED1B	CLK1	TM2D3	CLK4
	EIF5	HIST1H4C	HIST1H4C	WBP4	MBNL2	S1PR1
	TOP1	HIST2H2AC	S1PR1	MED19	PIP5K1B	LYPLAL1
	RARRES3	SKIL	MRPL18	DYNLL1	RNASE6	LOC102606465
	GIMAP4	TMEM11	HIST2H2AC	RPS24	RGCC	TAGAP
		ID3	SNORD13	ID3	ZNF107	LOC100294145
		RBM25	PFKFB3	RNF44	ZNF706	TMEM156
		IVNS1ABP	ID3	CHCHD1	CAPN10-AS1	MTMR10
		OSER1	LOC100289230	EVI2B	TM9SF2	MRFAP1
			MED26	MALSU1	TAOK1	ZC3H10
			RASSF3	NUCKS1	SELK	
			CLK4	RNA45S5	DNAJB9	
			LIPE	TNRC6A	TMEM147	
				ERICH1	IDNK	
				TRMT112	PDK1	
				MRPL18	RNF103	
				TRAPPC2B	NDUFB4	
				ATP5G2	RNF181	
				SSB	ST7	
				NAP1L1	HBP1	
				DGUOK	SPCS1	
				UQCR10	KRTCAP2	
				RCOR1	TVP23B	
					ALG2	
					SEC61B	
					MANEA	
					FANCF	

NK	CD16pMo	CD16nMo	pDC	mDC	Neu
CLK4	CLK1	HIST1H2AC	HIST1H4C	ZNF12	PARGP1
CLK1	MECP2	CLK1	RBX1	HIST1H2AC	CCNK
EIF5	FAM96B	APOBR	TMEM165	HIST1H4C	CCDC85B
	SKIL	HIST1H4C	FANCF	HIST1H4E	PPTC7
	HIST1H4C	RBM25	TNRC6A	ZNF436-AS1	AASDHPPT
	TM2D3	RSRP1	UBE2W	UBE2W	KIAA1919
	NDUFB1	KIAA0430	ZNF33B	SIAH1	CYCS
	CLK4	CXorf21	SNORD13	HYLS1	KIF13B
	CHORDC1	KLHL9	MARCH1	HIST1H2BG	FNBP4
	GOLGA4	ATM	RBM7	CLK1	BCCIP
	RSRP1	LRP1	SCARNA9	CYTIP	SDAD1
	NDUFB4	RENBP	TAF1A	LOC102724814	SMARCC1
	UQCR10	OSER1	DYNLL1	HIST1H2AD	KAT6A
	PURA	TAGAP	TOB2	KCTD7	MRPL13
	MED18	PEAR1	COMMD6	FADD	TFB2M
	HIST1H4E	PRPF38B	CLK1	ASF1A	KIAA1279
	LUC7L3	TOP1	ATPIF1	MAX	SDCCAG8
	FBRS	HIST2H2AC	HIST2H2AC	WDR75	NCBP1
	SNRPG	TRIM13	ZMYM6NB	LOC100289230	HAUS3
	NFKBID	RASSF3	NOP10	TAF1C	LSM4
	RSRC2	KMT2E	ARID3A	ZUFSP	ADK
	SETD1B	DDX17	LENG1	LUC7L3	LARP4
	ZNF396	MAX	NDUFB4	PHF23	ANKH
	EPOR	NDUFC1	CCDC186	HNRNPU-AS1	LZTFL1
	MED31	POU2F1	LOC652276	ZBTB11	ERC1
	FNBP4	PRRC2C	CKS2	DKFZP434I0714	LARS
	MAX	LUCAT1	PRDX1	HIST2H2AC	ZNF3
	KLHL9	AP1S2	RCOR1	RPL32P3	UBE2Q2
	LTB	CYP27A1	VTRNA1-1	COMMD3	RPL6
	COX7C	HIF1A	S100A11	HIST1H2BN	RABGAP1L
