# Supplementary material for: Transcriptome analysis of immune cells from Behçet’s syndrome patients: the importance of IL-17-producing cells and antigen-presenting cells in the pathogenesis of Behçet’s syndrome
Source: Arthritis Res Ther. 2022 Aug 8;24:186. doi: 10.1186/s13075-022-02867-x (PMC9358821; doi:10.1186/s13075-022-02867-x)
Supplement: Supplementary file 6 — Additional file 6. Genes downregulated in BS patients in each cell subset. [file 13075_2022_2867_MOESM6_ESM.pdf]

Additional file 6. Genes downregulated in BS patients in each cell subset

Top 30 genes (in the order of p values) downregulated in each cell subset. For subsets with less than 30 genes with FDR<0.05, only genes with FDR<0.05 are shown.

Naive CD4	Mem CD4	Th1	Th2	Th17	Tfh	Fr II eTreg
CLK1	LITAF	CLK1	DYNLL1	LINC00674	CLK1	CLK1
CLK4	CLK1	GIMAP2	CXCL5	CLK4	GIMAP7	CLK4
	MYBPC2	C5orf56	TM2D3	CLK1	C5orf56	LTB
	GIMAP2	CLK4	GIMAP7	RGCC	GIMAP2	RGCC
	CLK4	LINC00892	HIST1H4C	SNRPG	CLK4	C8orf59
	GZMA	RGCC	CLK1	LINC00493	NDUFC1	SCARNA9
	REEP4	SLIRP	GIMAP2	NDUFB1	POLR1C	DYNLT1
	GIMAP7	C12orf65	CKS2	MRPL18	GPR18	MED7
	RGCC	GIMAP7	C5orf56	EVL	NDUFB1	PCED1A
	JMJD6	CNIH4	CLK4	SH2B1	COX7B	TAGAP
	HIST2H2AC	C8orf59	RGCC	SNHG10	DPM1	LINC00493
	RBM48	C1orf52	RBX1	C8orf59	ABHD3	TRIM13
	CMC1	LINC00493	RARRES3		SNRPC	MAX
	EFHD2	SNRPG	OTUB1		UQCRH	CASP1P2
	RARRES3	C14orf119	MED19		MRPL39	ZNF14
	PRR5L	NDUFC1	BTG3		TMEM126B	DHRS1
	HCST	UQCR10	FAM96B		C11orf31	CARD16
	TMEM41B	SNORD13	NDUFB3		LINC00493	C5orf56
	DHRS1	KLHDC3	ATPIF1		RBX1	ID3
	KBTBD3	MAF	RAB33A		UBL5	COMMD6
	USP28	NDUFB1	SCARNA21		CHORDC1	KBTBD3
	GZMM	TNRC6A	CMC1		SNRPG	ZMYM6NB
	LINC00944	COX7A2	CD247		SRSF2	RNF44
	CD81	POLR2F	PLAC8		C12orf57	CIR1
	RPL32P3	EIF1B			MRPL36	CD3D
	KMT2E	MTRNR2L2			SLIRP	SNORD13
	TGIF1	IZUMO4			CARD16	C12orf65
	SNRPG	PET100			KBTBD3	RPP38
	CST7	ATPIF1			CMC1	TMEM60
	FASLG	LOC102606465			NOP10	LRRC16A

Naive CD8	Mem CD8	Naive B	USM B	SM B	Plasmablast	DN B
None	HIST1H4C	CLK1	CLK1	HIST1H4C	INSIG2	CLK1
	CLK1	MED21	RPL32P3	SNORD13	FAM200A	ID3
	DPM1	HBP1	NOP10	NOP10	HERPUD1	P2RY10
	PIGF	CLK4	PCED1B	CLK1	TM2D3	CLK4
	EIF5	HIST1H4C	HIST1H4C	WBP4	MBNL2	S1PR1
	TOP1	HIST2H2AC	S1PR1	MED19	PIP5K1B	LYPLAL1
	RARRES3	SKIL	MRPL18	DYNLL1	RNASE6	LOC102606465
	GIMAP4	TMEM11	HIST2H2AC	RPS24	RGCC	TAGAP
		ID3	SNORD13	ID3	ZNF107	LOC100294145
		RBM25	PFKFB3	RNF44	ZNF706	TMEM156
		IVNS1ABP	ID3	CHCHD1	CAPN10-AS1	MTMR10
		OSER1	LOC100289230	EVI2B	TM9SF2	MRFAP1
			MED26	MALSU1	TAOK1	ZC3H10
			RASSF3	NUCKS1	SELK	
			CLK4	RNA45S5	DNAJB9	
			LIPE	TNRC6A	TMEM147	
				ERICH1	IDNK	
				TRMT112	PDK1	
				MRPL18	RNF103	
				TRAPPC2B	NDUFB4	
				ATP5G2	RNF181	
				SSB	ST7	
				NAP1L1	HBP1	
				DGUOK	SPCS1	
				UQCR10	KRTCAP2	
				RCOR1	TVP23B	
					ALG2	
					SEC61B	
					MANEA	
					FANCF	

NK	CD16pMo	CD16nMo	pDC	mDC	Neu
CLK4	CLK1	HIST1H2AC	HIST1H4C	ZNF12	PARGP1
CLK1	MECP2	CLK1	RBX1	HIST1H2AC	CCNK
EIF5	FAM96B	APOBR	TMEM165	HIST1H4C	CCDC85B
	SKIL	HIST1H4C	FANCF	HIST1H4E	PPTC7
	HIST1H4C	RBM25	TNRC6A	ZNF436-AS1	AASDHPPT
	TM2D3	RSRP1	UBE2W	UBE2W	KIAA1919
	NDUFB1	KIAA0430	ZNF33B	SIAH1	CYCS
	CLK4	CXorf21	SNORD13	HYLS1	KIF13B
	CHORDC1	KLHL9	MARCH1	HIST1H2BG	FNBP4
	GOLGA4	ATM	RBM7	CLK1	BCCIP
	RSRP1	LRP1	SCARNA9	CYTIP	SDAD1
	NDUFB4	RENBP	TAF1A	LOC102724814	SMARCC1
	UQCR10	OSER1	DYNLL1	HIST1H2AD	KAT6A
	PURA	TAGAP	TOB2	KCTD7	MRPL13
	MED18	PEAR1	COMMD6	FADD	TFB2M
	HIST1H4E	PRPF38B	CLK1	ASF1A	KIAA1279
	LUC7L3	TOP1	ATPIF1	MAX	SDCCAG8
	FBRS	HIST2H2AC	HIST2H2AC	WDR75	NCBP1
	SNRPG	TRIM13	ZMYM6NB	LOC100289230	HAUS3
	NFKBID	RASSF3	NOP10	TAF1C	LSM4
	RSRC2	KMT2E	ARID3A	ZUFSP	ADK
	SETD1B	DDX17	LENG1	LUC7L3	LARP4
	ZNF396	MAX	NDUFB4	PHF23	ANKH
	EPOR	NDUFC1	CCDC186	HNRNPU-AS1	LZTFL1
	MED31	POU2F1	LOC652276	ZBTB11	ERC1
	FNBP4	PRRC2C	CKS2	DKFZP434I0714	LARS
	MAX	LUCAT1	PRDX1	HIST2H2AC	ZNF3
	KLHL9	AP1S2	RCOR1	RPL32P3	UBE2Q2
	LTB	CYP27A1	VTRNA1-1	COMMD3	RPL6
	COX7C	HIF1A	S100A11	HIST1H2BN	RABGAP1L
