# Supplementary material for: Transcriptome analysis of immune cells from Behçet’s syndrome patients: the importance of IL-17-producing cells and antigen-presenting cells in the pathogenesis of Behçet’s syndrome
Source: Arthritis Res Ther. 2022 Aug 8;24:186. doi: 10.1186/s13075-022-02867-x (PMC9358821; doi:10.1186/s13075-022-02867-x)
Supplement: Supplementary file 7 — Additional file 7. MA plot of each cell subset. [file 13075_2022_2867_MOESM7_ESM.pdf]

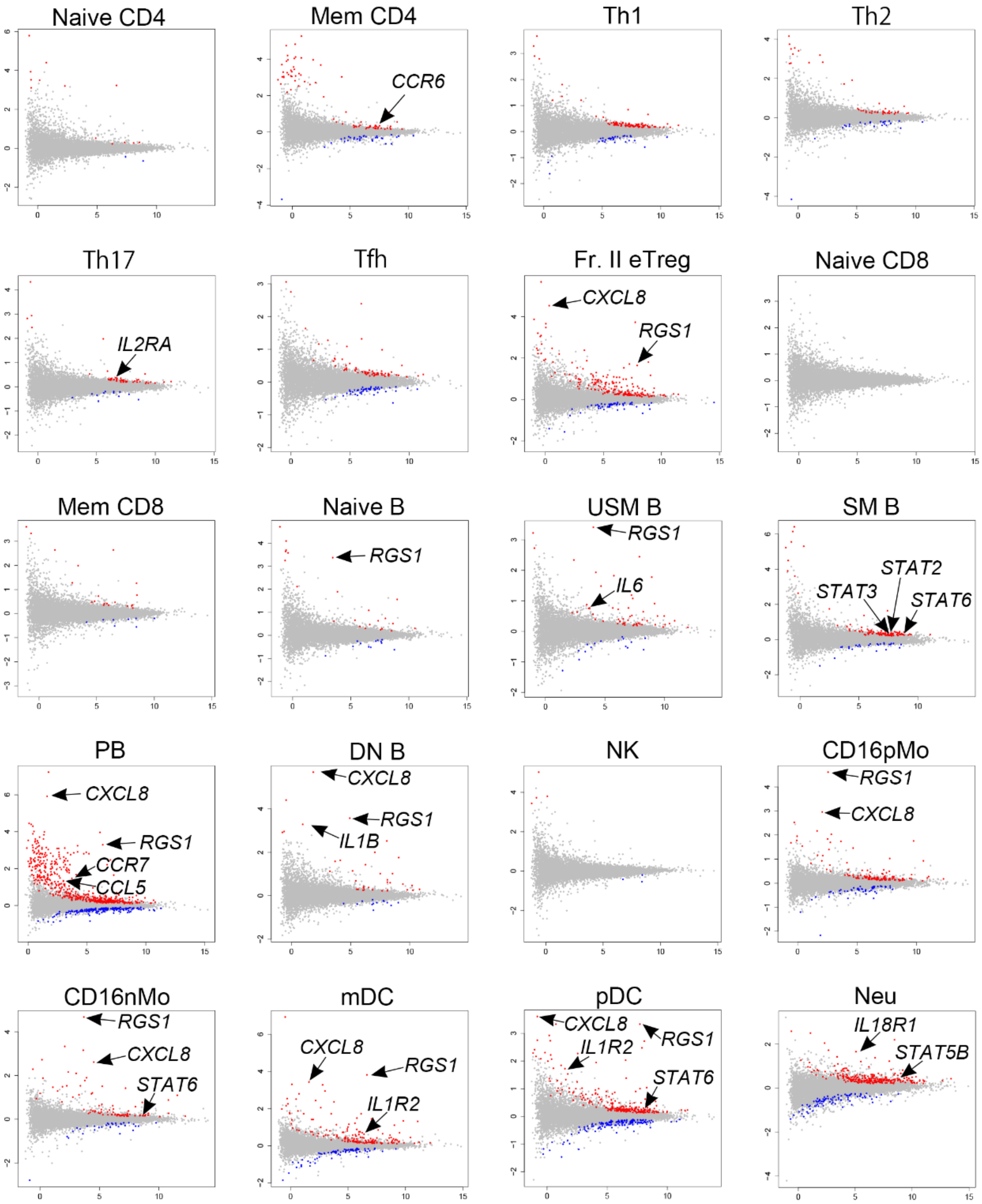

Additional file 7. MA plot of each cell subset

MA plots with immunologically important DEG highlighted
