# Supplementary figures and images for: Transcriptome analysis of immune cells from Behçet’s syndrome patients: the importance of IL-17-producing cells and antigen-presenting cells in the pathogenesis of Behçet’s syndrome
Source: Arthritis Res Ther. 2022 Aug 8;24:186. doi: 10.1186/s13075-022-02867-x (PMC9358821; doi:10.1186/s13075-022-02867-x)

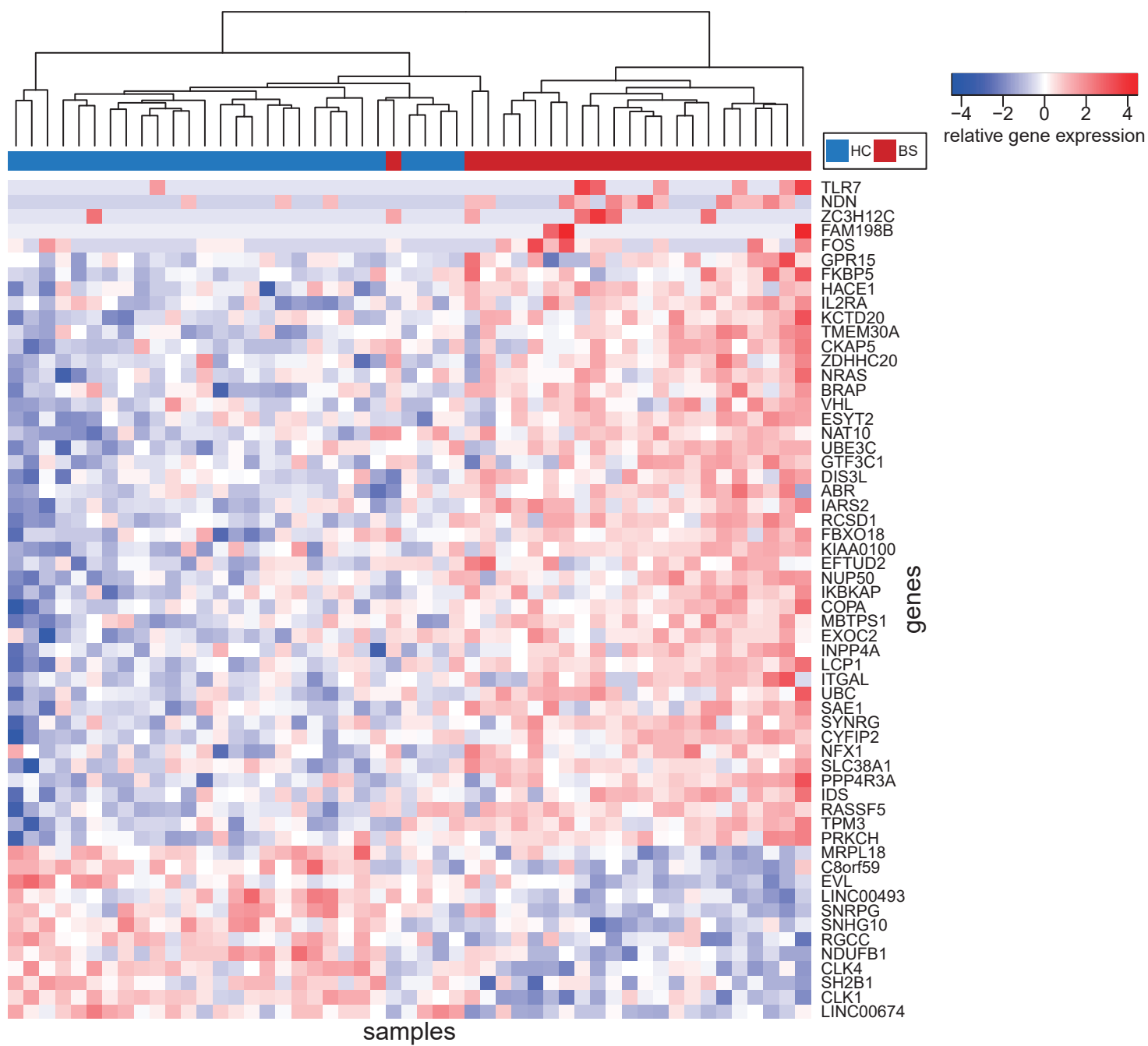

**Additional file 8. Heatmap of DEG in Th17 cells**

Supplement: Supplementary file 8 — Additional file 8. Heatmap of DEG in Th17 cells. [file 13075_2022_2867_MOESM8_ESM.pdf]
