# Supplementary material for: Transcriptome analysis of immune cells from Behçet’s syndrome patients: the importance of IL-17-producing cells and antigen-presenting cells in the pathogenesis of Behçet’s syndrome
Source: Arthritis Res Ther. 2022 Aug 8;24:186. doi: 10.1186/s13075-022-02867-x (PMC9358821; doi:10.1186/s13075-022-02867-x)
Supplement: Supplementary file 9 — Additional file 9. Pathway Analysis of DEG between BS Patients. [file 13075_2022_2867_MOESM9_ESM.pdf]

Additional file 9. Pathway Analysis of DEG between BS Patients and healthy controls in Th17 cells

Pathway	-log(p-value)	Ratio
Renal Cell Carcinoma Signaling	4.06E+00	4.65E-02
NF-κB Activation by Viruses	3.92E+00	4.30E-02
UVC-Induced MAPK Signaling	3.49E+00	6.12E-02
Systemic Lupus Erythematosus Signaling	3.46E+00	2.24E-02
IL-2 Signaling	3.04E+00	4.29E-02
Thrombopoietin Signaling	3.02E+00	4.23E-02
Toll-like Receptor Signaling	2.95E+00	4.00E-02
Erythropoietin Signaling	2.77E+00	3.45E-02
IL-3 Signaling	2.74E+00	3.37E-02
Prolactin Signaling	2.74E+00	3.37E-02
NRF2-mediated Oxidative Stress Response	2.70E+00	2.03E-02
