# Supplementary material for: Transcriptome analysis of immune cells from Behçet’s syndrome patients: the importance of IL-17-producing cells and antigen-presenting cells in the pathogenesis of Behçet’s syndrome
Source: Arthritis Res Ther. 2022 Aug 8;24:186. doi: 10.1186/s13075-022-02867-x (PMC9358821; doi:10.1186/s13075-022-02867-x)
Supplement: Supplementary file 10 — Additional file 10. Top 20 members of modules with significant correlation with clinical parameters. [file 13075_2022_2867_MOESM10_ESM.pdf]

Additional file 10. Top 20 members of modules with significant correlation with clinical parameters

Top 20 members of modules with absolute correlation coefficient > 0.4 and p-values < 0.05 are shown.

Naive B_06	Naive B_07	SM B_01	SM B_02	SM B_06	SM B_07	SM B_08	SM B_10	SM B_12	SM B_14
DUSP1	ARHGDIA	XIST	RPL35A	DNAJC10	RNF126	FAU	COX5B	TGFB1	RAD23A
FOS	ATP6V0C	EIF1AX	RPL26	RBL2	TSR3	RPL35	POLR2L	SPI1	TMUB1
PPP1R15A	MKNK2	ZRSR2	RPS13	PIK3AP1	ADRM1	RPL36	ATP5I	ARHGDIA	LYL1
FOSB	SPI1	RPS4X	RPL32	ANKRD13A	ATP5D	RPS15	C19orf53	ATP6V0C	ARL2
JUNB	TGFB1	TXLNG	RPL11	PPP4R3B	CCDC85B	RPS19	OST4	FKBP8	LIMD2
NR4A2	WAS	JPX	PFDN5	ADAM10	FAM173A	RPL18	RPL18A	MKNK2	RPL28
RGS1	MAZ	KDM5C	RPL39	CLTC	NDUFS7	C11orf31	POLR2I	SF3A2	ATP5E
DUSP2	FKBP8	ZFX	RPS20	EIF4G2	NUBP2	RPL13	PYCARD	SEPT9	PTOV1
KLF6	SF3A2	EIF2S3	RPS8	ERBB2IP	SCAND1	RPS28	TCEB2	GNB2	BCL7C
CD69	GNB2	KDM6A	RPL10A	HIPK3	SPSB3	SIVA1	NAA38	TSC22D4	FAAP20
EGR1	PPDPF	PRKX	RPL24	MSN	SSNA1	LAMTOR4	SERF2	MED25	IRF3
UBC	RAVER1	DDX3X	RPL27	PIKFYVE	AURKAIP1	PNKD	TMSB10	WAS	SMG1P5
JUN	SEPT9	CXorf38	RPL38	KIDINS220	C19orf60	RPL13A	UQCR11	SMARCB1	RUNX3
JUND	TSC22D4	OFD1	RPS11	LRPPRC	CORO1A	RPL19	LINC00116	PNPLA2	C6orf1
HES1	GPSM3	SYAP1	RPL21	MATR3	CYBA	RPL29	NDUFA3	RGS19	MRPS2
IER2	MED25	DLEU1	RPL30	SCRN1	GADD45GIP1	TRMT112	RABAC1	DGKZ	RHOF
MOAP1	MTA1	HDHD1	RPL34	ZDHHC20	MRPL41	CUTA	COX8A	MTA1	MZT2B
MIR616	RNPEPL1	CASP8AP2	RPL37	API5	OAZ1	EEF1D	LAMTOR2	PQLC1	ARPC4
RGS2	CD37	LOC389906	RPS15A	NCOA3	PTPRCAP	EIF3G	NDUFB4	RABL6	C18orf32
SOCS3	KLF2	PNPLA4	RPL31	PHKB	TPGS1	LSM7	C4orf48	RAVER1	FLT3LG

USM B_01	USM B_04	USM B_07	USM B_08	USM B_09	DN B_03	DN B_06	DN B_08	DN B_10	DN B_12
XIST	UQCR10	CNOT1	DUSP1	SPI1	AKAP13	ALOX5	TCEB2	XIST	DUSP1
KDM5C	COX5B	DOCK2	NR4A2	ARHGDIA	ATP8A1	CD40	COX5B	ZFX	PPP1R15A
PRKX	COX6A1	RASSF2	RGS1	RAVER1	PIKFYVE	FADS3	EDF1	KDM5C	FOS
CXorf38	UQCR11	PRPF8	FOS	ATP6V0C	CDK14	HVCN1	NDUFA13	EIF1AX	JUNB
KDM6A	TRMT112	LCP1	DUSP2	MKNK2	MEF2A	GYLTL1B	NDUFB7	KDM6A	KLF6
EIF1AX	CD52	PIK3AP1	FOSB	SEPT9	RAPGEF6	FAM129C	RPS15	DDX3X	FOSB
JPX	COX6B1	SNRNP200	PPP1R15A	TSC22D4	LRRK2	PNRC1	NDUFA11	JPX	NR4A2
ZFX	FAM96B	MSN	JUNB	GNB2	DOCK10	ZSCAN18	NDUFS7	EIF2S3	NFKBIA
EIF2S3	MYEOV2	TRIP12	RGS2	MED25	RPS6KA3	MARCKSL1	ROMO1	ZRSR2	DUSP2
SYAP1	SNRPD2	PPP1R16B	EGR1	TGFB1	WDFY4	POLD4	ANAPC11	SMC1A	RGS2
ZRSR2	MEA1	RBL2	MIR616	WAS	GPCPD1	VPREB3	POLR2L	CXorf38	EIF1
TXLNG	UBL5	LRPPRC	ZFP36	FKBP8	PTK2	CCDC151	AURKAIP1	USP9X	RGS1
DDX3X	ATP5I	CSDE1	CD69	PNPLA2	ATL3	NOXA1	GADD45GIP1	CA5BP1	ZFP36
SMC1A	COMMD6	PUM1	SOCS3	SH3GL1	ATP10D	SYBU	GUK1	HDHD1	EGR1
HDHD1	COX17	COPA	JUN	VASP	PLXNC1	CCDC106	MPG	TXLNG	JUN
RPS4X	COX7C	DDB1	KLF6	GPSM3	RASSF2	CCR7	MRPL41	PRKX	CD69
CA5BP1	KRTCAP2	EIF4G2	HES1	MAP3K11	ST6GAL1	COCH	OAZ1	LOC389906	CSRNP1
USP9X	NDUFB2	MAT2A	OTUD1	TBC1D10B	TGFBR2	RRP7BP	SNRPB	STAT5B	AREG
TSIX	ATP5L	VPS39	EIF1	CDC37	CORO1C	CIRBP	CYBA	RPS4X	ARL4A
INE1	COX7A2	DHX9	IER2	DMWD	IKZF1	CXCR5	NAA38	SEPT6	PMAIP1

DN B_13	Plasmablast_05	Plasmablast_16	Th1_02	Th1_13	Th2_09	Th17_05	Th17_06	Th17_08	Tfh_03
PIK3CD	OSTC	PRPF8	POLR2L	XIST	NPM1	TYROBP	CCDC85B	ARHGDIA	RORA-AS1
STK10	DPM1	DOCK2	C19orf24	EIF1AX	RPL7	CPVL	TMUB1	MKNK2	ASH1L
INPP5D	SUB1	PARP4	NDUFA3	PRKX	BTF3	MNDA	CTU1	SEPT9	MGEA5
DCTN1	DAD1	SNRNP200	C4orf48	KDM5C	PSIP1	SPI1	RNF126	TSC22D4	ANKRD36
MYH9	DYNLL1	MYH9	HSPB1	EIF2S3	EIF3E	FCER1G	SSBP4	WAS	RUNX1-IT1
TCOF1	MPC1	SPTAN1	RAD23A	ZFX	NACA	FCN1	STUB1	ATP6V0C	SERINC5
CIITA	B2M	TRIP12	RNF126	KDM6A	HNRNPA1	FGR	FBXL15	FKBP8	UTRN
PHRF1	SEP15	CNOT1	SSBP4	RPS4X	SKP1	LILRB2	PDLIM2	TGFB1	KMT2A
FLNA	LAMTOR5	COPA	TMUB1	SEPT6	ESD	LRRC25	TMEM160	ZBTB7B	ZBTB20
PPP6R1	TCEB1	CRKL	ATP5D	ZRSR2	SSB	MPEG1	C4orf48	CBX6	ANKRD44-IT1
SUN2	GABARAPL2	KCTD20	FAM195B	LOC389906	RSL1D1	CST3	CCDC107	GNB2	LOC100506860
TBC1D9B	HINT1	SPTBN1	RPL28	JPX	HMGN1	HCK	MRPS34	GPSM3	SYNE2
CEP250	MRPL50	CAPRIN1	CTU1	CA5B	PA2G4	HK3	TSR3	IRF2BPL	TNRC6B
INTS1	POLR2K	DOCK8	RPS28	TXLNG	CCDC59	IFI30	ZBTB7A	EPN1	ANKRD36C
NUP210	REEP5	DDB1	ARL2	CXorf38	NAP1L1	SERPINA1	SIGIRR	DGKZ	ERC1
VAV2	RPS27L	IQGAP1	C9orf16	DDX3X	BRIX1	CD68	C20orf24	NELFB	NKTR
MYO18A	SFT2D1	PI4KA	KLF2	CA5BP1	EEF1A1	CLEC12A	DBP	RABL6	OGT
NUMA1	ZNF706	STAG1	LIMD2	AP1S2	LDHB	LYN	FAAP20	RAVER1	PSMD6-AS2
TLN1	C8orf59	ATP10D	ZBTB7A	HDHD1	SYF2	S100A8	RBM42	SF3A2	RALGAPA2
ARHGAP27	SPCS1	KIAA0100	BCL7C	SMC1A	CDC26	CD300E	SHARPIN	MAP7D1	ATXN7

Tfh_04	Naive CD4_01	Naive CD4_04	Naive CD4_07	Mem CD4_01	Mem CD4_06	Mem CD4_08	Mem CD4_12	Mem CD4_14	Mem CD4_15
LRCH4	TCEB2	ARHGDIA	XIST	ARHGDIA	TCEB2	EDF1	C19orf60	XIST	RPL7
TMEM259	DRAP1	TSC22D4	EIF1AX	ATP6V0C	ANAPC11	ATP5D	DPP7	KDM5C	RPL41
RHOT2	EDF1	FKBP8	KDM5C	MKNK2	NDUFS7	CYBA	SPSB3	ZRSR2	RPS6
ARHGAP4	MRPS34	ATP6V0C	KDM6A	DGKZ	COX5B	PIN1	TBC1D10C	EIF1AX	TPT1
SYTL1	RNF126	SEPT9	LOC389906	SEPT9	NDUFA11	C9orf16	DUS1L	PRKX	BTF3
ARHGEF1	STUB1	DGKZ	PRKX	WAS	NDUFA13	DRAP1	SYTL1	KDM6A	NPM1
HEXDC	ATP5D	PQLC1	ZRSR2	TGFB1	NDUFB7	FAM89B	TMEM259	ZFX	EIF3E
TBC1D10C	AURKAIP1	MKNK2	JPX	TSC22D4	COPE	POLR2L	RASSF7	SEPT6	RPL10A
DPP7	CCDC85B	KLF2	SEPT6	VASP	OAZ1	SSBP4	RHOT2	EIF2S3	RPL21
DUS1L	GADD45GIP1	SF3A2	EIF2S3	ZBTB7B	PHPT1	TMEM160	TCIRG1	JPX	RPL9
ACAP1	NDUFA11	WAS	TXLNG	NELFB	APRT	AURKAIP1	ACAP1	TXLNG	RPS15A
BRAT1	NDUFS7	ATP2A3	CA5BP1	ZYX	SSNA1	STUB1	ARHGAP4	DDX3X	RPS23
MUS81	OAZ1	GNB2	ZFX	FKBP8	TBCB	CCDC85B	GUK1	CA5BP1	EEF1B2
TCIRG1	PIN1	H1FX	RPS4X	MESDC1	UQCR11	LIME1	PPP1R35	RPS4X	RPL39
ZAP70	C9orf16	NELFB	DDX3X	RABL6	LAMTOR4	PTPRCAP	FASTK	HDHD1	RPL5
FBXW5	NDUFS8	PNPLA2	SYAP1	SMARCB1	ROMO1	ADRM1	FBXL15	SYAP1	RPL6
RASSF7	SCAND1	RABL6	CXorf38	ATP2A3	SSR4	FAM195B	VPS28	LOC389906	NACA
FASTK	SPSB3	CD37	HDHD1	GPSM3	ATP6V1F	NAA38	RGS14	CXorf38	RPL24
RASAL3	TMEM160	IRF2BPL	PNPLA4	RELA	MRPL41	TMUB1	SGSM3	AP1S2	RPSA
TRABD	C19orf24	GPSM3	SMC1A	GNB2	MRPS34	CTU1	ARHGEF1	UBA1	RPL32

Fr. II eTreg_01	Fr. II eTreg_02	Fr. II eTreg_04	Naive CD8_02	Naive CD8_04	Naive CD8_06	Naive CD8_08	Mem CD8_08	Mem CD8_09	Mem CD8_10
DUSP1	TCEB2	RRM2	POLR2L	MYC	MYO1F	SSR4	RORC	ARHGDIA	XIST
DUSP2	COX5B	STMN1	TMEM160	SATB1	PRF1	COX6A1	CEBPD	FKBP8	EIF1AX
PPP1R15A	ZNHIT1	ZWINT	C4orf48	ACTN1	APMAP	CHCHD2	COLQ	DGKZ	EIF2S3
FOSB	NDUFS6	DHFR	TCEB2	CCR7	CALHM2	MYEOV2	SLC4A10	MKNK2	KDM6A
JUNB	POLR2L	MCM7	C20orf24	DGKA	CCL5	PSMA7	TSPAN15	TGFB1	ZRSR2
FOS	UQCR11	TUBA1B	C9orf16	NT5E	CLIC1	PSMB3	IL23R	TSC22D4	TXLNG
JUN	NDUFA11	HMGB2	EDF1	ABLIM1	CST7	RPL36AL	SCRN1	SEPT9	DDX3X
NR4A2	CYBA	UHRF1	HCST	AMICA1	DOK2	COX8A	TMIGD2	ATP6V0C	KDM5C
PMAIP1	LSM7	CCNB1	ATP5D	FAM117B	EFHD2	KRTCAP2	CCR6	NELFB	SEPT6
CD69	NDUFA13	CCNB2	C19orf70	LEF1	GZMH	MYL6	KLRB1	RAVER1	PRKX
RGS1	RPLP1	DLGAP5	DRAP1	RCAN3	NKG7	NDUFA6	NRIP1	GNB2	HDHD1
BTG2	C9orf16	FEN1	FAM195B	BACH2	SLC9A3R1	PSMB6	ABCB1	MED25	JPX
DNAJB1	PRDX5	KIAA0101	PPIB	FAM102A	STOM	S100A6	CXXC5	WAS	LOC389906
NFKBIA	PTPRCAP	PCNA	PTPRCAP	LDLRAP1	APOBEC3C	ATP6V1F	FLT4	KLF2	ZFX
ZFP36	ROMO1	TPX2	RABAC1	CAMK4	CTSC	SEC61B	IL4I1	ZNF598	RPS4X
PER1	ANAPC11	TUBB	STUB1	CHMP7	F2R	SNRPB	LTK	CAPN15	CA5B
RGS2	C19orf53	TYMS	CCDC107	DENND5A	FGR	TMSB4X	ME1	EPN1	CA5BP1
BRE-AS1	DRAP1	ASF1B	COPE	EPHX2	GPR68	CHMP2A	ZBTB16	BBC3	CXorf38
MIR616	NDUFS8	BIRC5	CYBA	FOXO1	LPCAT1	DBI	P2RY14	PKN1	MSL3
UBC	TMSB10	TK1	GUK1	IL6R	PLEKHF1	EIF3K	TLE1	RPS6KA4	AP1S2

Mem CD8_11	Mem CD8_13	mDC_01	mDC_04	mDC_06	mDC_07	mDC_09	mDC_10	pDC_01	pDC_06
RNF126	PF4	IFI44L	RPS15	SEPT9	CADM1	DUSP1	CA2	IFI44L	FAU
ATP5D	PPBP	IFIT3	TCEB2	ARHGDIA	DNASE1L3	FOS	DHRS9	MX1	UQCR11
C19orf24	GNG11	OAS2	NDUFA13	SPI1	IRF8	PPP1R15A	ARHGAP17	XAF1	FKBP2
CCDC85B	F13A1	IFI6	ANAPC11	TGFB1	RAB7B	RGS1	CD180	IFI44	NDUFS6
FAM195B	GP9	IFI44	CYBA	VASP	GCSAM	RGS2	ECHDC1	ISG15	POLR2L
RAD23A	ITGB3	IFITM1	EDF1	EPN1	CLEC9A	CXCL8	ALAS1	OAS2	PYCARD
TMEM160	SPARC	CMPK2	NDUFA11	FKBP8	PNMA1	CHMP1B	ADRB2	EIF2AK2	COX6A1
TMUB1	SDPR	HERC5	NDUFB7	WAS	CLNK	CXCR4	C3AR1	PLSCR1	COX8A
ZBTB7A	ITGA2B	IFIT1	POLR2L	ATP6V0C	CPNE3	OTUD1	TLR2	SAMD9L	NAA38
C9orf16	TUBB1	MX1	RPL18A	DGKZ	TACSTD2	PMAIP1	FAM107B	USP18	RPL35
CTU1	NRGN	PARP9	RPL36	GNB2	C1orf54	DUSP2	LCP2	EPSTI1	RPL36
CYBA	TREML1	EIF2AK2	TIMM13	INPPL1	IDO1	ARL4A	HVCN1	STAT1	TCEB2
DRAP1	C6orf25	IFIT2	AURKAIP1	RPS6KA4	SNX3	CSRNP1	EPS8	LAP3	COX5B
FAM89B	PTGS1	SIGLEC1	FAU	ZBTB7B	XCR1	KLF4	UBE2J1	UBE2L6	LSM7
PDLIM2	TRIM58	XAF1	GUK1	NELFB	RGCC	NR4A2	RUNX2	PARP12	NDUFA11
PIN1	PRKAR2B	OAS3	LAMTOR4	RABL6	SNX22	UBC	CD53	CMPK2	NDUFA13
POLR2L	SMOX	OASL	OAZ1	RAVER1	ENOX1	YRDC	IGSF6	HESX1	NDUFA7
STUB1	CXCL5	EPSTI1	PIN1	TSC22D4	APOL1	ID1	NFKB1	IFI16	RPS15
ARL2	C2orf88	LY6E	APRT	HMGA1	BTLA	ZFP36	ACTR3	IFI35	FAAP20
CSNK1G2	ABCC3	ISG15	C11orf31	ARL8A	KIAA0226L	FOSB	ALCAM	LY6E	RPL18A

pDC_07	pDC_13	pDC_15	pDC_16	CD16p Mono_01	CD16p Mono_02	CD16p Mono_03	CD16p Mono_05	CD16p Mono_07	CD16n Mono_01
COTL1	TSR3	RGS2	POMP	ARHGDIA	PPP1R15A	C4orf48	IFI44L	NDUFA13	ARPC3
ABI3	CCDC85B	FOS	ARPC3	SPI1	DUSP1	FKBP2	USP18	RPS15	ATP5F1
IFI30	MXD4	DUSP1	ATP5L	ATP6V0C	FOS	LIMD2	IFIT1	C19orf70	DPY30
CEBPA	ATP5D	RGS1	DYNLL1	EPN1	FOSB	POLR2L	IFIT3	EDF1	SRP14
FGR	SLC52A2	FOSB	HINT1	GNB2	ZFP36	RNF126	OASL	NDUFA11	ATP5O
CD22	COPE	AREG	NDUFA4	SEPT9	RGS1	LYL1	IFI44	ROMO1	BTF3
CX3CR1	FAM89B	JUNB	RBX1	WAS	G0S2	C20orf24	RSAD2	TCEB2	NACA
ALOX5	NDUFS7	ZFP36	LAMTOR5	VASP	NR4A2	CCDC107	CMPK2	COX8A	NDUFA4
BASP1	PIN1	PMAIP1	NDUFV2	RXRA	CCL3L1	FAAP20	IFIT2	FAU	PFDN5
TIMP1	AURKAIP1	LOC100289511	SUB1	TGFB1	JUN	PYCARD	EPSTI1	NDUFB7	RPL41
ANXA1	DPP7	SOCS3	COX6C	NELFB	BRE-AS1	RBM42	XAF1	RPL18A	SKP1
CEBPD	GADD45GIP1	JUN	HSPB11	ZBTB7B	NFKBIA	SSBP4	OAS2	RPL35	CWC15
CLEC10A	JMJD8	NR4A3	PSMA2	CAPN15	CXCL8	ADRM1	OAS3	TYROBP	NDUFB3
ID2	LIME1	CXCL2	CKS1B	FKBP8	KLF6	ATP5D	SIGLEC1	CISD3	PSMB1
SIGLEC1	MAF1	PPP1R15A	LSM1	PNPLA2	OTUD1	C19orf24	EIF2AK2	COX5B	ENY2
ADAM33	MRPS34	ARL4A	SF3B6	MAP3K11	LINC00936	DOK1	MX1	CYBA	PDCD6
FBLN2	NR1H2	NR4A2	SPCS1	MKNK2	CXCL2	FAM195B	IFI6	RPL36	SNAPIN
KLF4	NUBP2	ETV3	C14orf2	GRINA	JUNB	PIN1	TRIM22	ANAPC11	SPCS1
TNFAIP2	RBM42	DUSP6	NDUFB3	PPP2R4	LOC100289511	POLR2E	DDX58	LAMTOR4	TMSB4X
CFP	SCAND1	PLK2	PSMB1	CD37	RNU4ATAC	TMUB1	HERC5	NDUFA7	ATP6V0E1

CD16n Mono_04	CD16n Mono_05	CD16n Mono_07	Neu_03	Neu_05	Neu_06	Neu_09	Neu_10	Neu_11	Neu_13
SPI1	IFIT2	CYBA	EXOSC8	NCL	CD24	APMAP	SETX	BACE2	PLEKHM1
TGFB1	IFIT3	NDUFA3	NPM1	RPL13	CEACAM6	S100A12	OSTM1	ESYT1	CNOT3
ARHGDIA	CMPK2	POLR2L	SHPRH	RPL23A	CEACAM8	SRPK1	ATAD2B	GATA1	FKBP8
GNB2	EPSTI1	TIMM17B	BCCIP	ACP1	GFI1	ACSL1	BRWD1	ALOX15	MAP7D1
VASP	RSAD2	C4orf48	THAP7	ECI1	MS4A3	RAB7A	DDX6	PRSS33	MKNK2
TSC22D4	IFI44	COX8A	ZW10	RPL15	BPI	S100A9	PNRC2	PTGDR2	SF3A1
WAS	IFI44L	LIMD2	CUL5	CCT6A	NKG7	KLHL2	PRR14L	RFTN1	ABTB1
ATP6V0C	OASL	PYCARD	SRP68	L3MBTL2	DEFA4	PYGL	CEP170	SIGLEC8	GNB2
PNPLA2	IFIT1	RAD23A	TCERG1	PPIA	ELANE	DHRS7	NUFIP2	SLC29A1	ADRBK1
SEPT9	OAS2	RPL28	AKAP11	MRPS21	MPO	MBOAT2	GTF2A1	ADGRG5	TFE3
FKBP8	XAF1	RPLP2	MTDH	RPL3	AZU1	NRD1	ZNF721	FYN	TGFB1
ARL8A	DDX58	ATP5D	SLC25A36	RPL7L1	LAIR1	S100A8	NPAT	PIK3R6	CANT1
CAPN15	MX1	CST3	ZNF10	IMP3	OLFM4	CNN2	LTN1	PPP1R16B	RAB5B
DGKZ	PARP9	DRAP1	DPP7	RAD23A	OLR1	DYSF	RAB3GAP1	SMPD3	STXBP2
FAM78A	SAMD9L	NDUFS6	NOL8	EIF2B1	CTSG	HCLS1	SLC39A7	SPNS3	TNIP1
MKNK2	SIGLEC1	POLR2E	AARS2	HSPA9	SERPINB10	PHC2	MYSM1	ADGRE4P	ZSWIM8
RABL6	HSH2D	RPS28	BAZ1B	LSM4	ABCA13	TSPO	USO1	ARL4C	CAMTA2
RXRA	IFI6	FKBP2	EXOSC10	PA2G4	SFXN1	CST7	APAF1	HMGN3	GMIP
ZBTB7B	SERPING1	OST4	ICE2	RPL8	STMN1	EXOC6	ATM	ITGB7	LAMP1
EPN1	USP18	PIN1	KIAA1919	SEC31A	TOP2A	FLOT2	JARID2	PYROXD2	MAP3K11

Neu_15	Neu_18	Neu_19	Neu_20	Neu_21	Neu_24	NK_06	NK_07
CMPK2	AKNA	MYADM	LYSMD2	HLA-DMA	RAD23B	LAG3	GSN
IFIT3	RERE	PIGB	POLB	KARS	ERH	TTC16	FAM43A
RSAD2	C15orf39	EFHC1	RPAP3	APOL3	DDX17	PTMS	CCNJL
USP18	PPP1R18	ERCC1	PANK2	RFX5	RAB5C	RAP2A	CYSLTR1
BISPR	SF1	FAR2	PSME1	NOD1	WHAMM	MCOLN2	IL2RB
DDX60	VASP	RABGAP1L	CLEC2B	CD74	CTSZ	RAB11FIP5	CXXC5
IFI44L	KMT2D	ARMC2	NUB1	HLA-DMB	HNRNPA0	LILRB1	PDE6G
ISG15	PRRC2A	FUZ	MRPL44	SSB	CBX1	MSC	C1orf162
IFI44	TIMP2	SPOP	PSME2	GBP4	RBBP6	TRG-AS1	IL18RAP
OAS2	SETD1B	TSPAN2	PDE4B	C9orf91	ANP32A	VSTM2B	NEIL1
C19orf66	TNRC18	MTX1	STX11	IDO1	BAX	GDPD5	TMIGD2
IFIH1	MBD6	RIBC1	TNFAIP6	NFKBIE	BCAS2	CLEC2D	TTC9
IFIT1	PPP1R9B	SLC31A1	CXorf21	PSMB2	CEP295	KLRAP1	CHST2
PARP12	LPAR2	CFAP45	JAGN1	GBP5	CDKN1B	LGALS3	DHRS3
SPATS2L	ACTN4	FAM63A	TNFSF13B	MESDC2	RSL24D1	SGCB	LRRC70
TRIM5	BAG6	MOK	IRF2	AGPAT5	ERGIC1	FAS	ZSCAN18
ZBP1	FBRS	PHOSPHO1	MITD1	CASP7	RAP2C	LINC00944	FCER1G
HERC5	UBC	TAGLN2	PLAGL1	CBR1	STK16	RGS9	ZNF516
IFI6	BRI3	TMEM216	PSMA3	CNDP2	UBE2E1	ABCD2	SCRN1
IFIT2	CREBBP	SFR1	ATP6V1G1	MFSD12	DNAJC7	ARHGAP10	BEX2
