# Supplementary material for: Transcriptome analysis of immune cells from Behçet’s syndrome patients: the importance of IL-17-producing cells and antigen-presenting cells in the pathogenesis of Behçet’s syndrome
Source: Arthritis Res Ther. 2022 Aug 8;24:186. doi: 10.1186/s13075-022-02867-x (PMC9358821; doi:10.1186/s13075-022-02867-x)
Supplement: Supplementary file 12 — Additional file 12 eQTL effect of rs1518111 on IL10. [file 13075_2022_2867_MOESM12_ESM.pdf]

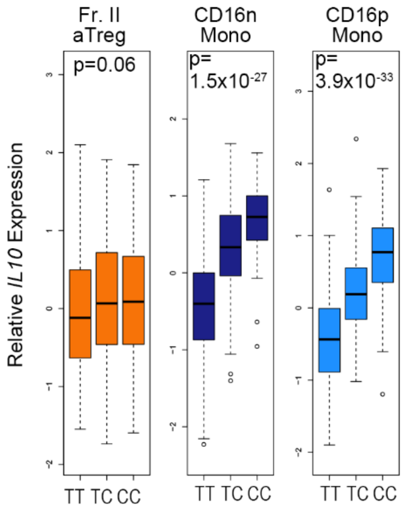

Additional File 12. eQTL effect of rs1518111 on *IL10*

The expression of *IL10* by rs1518111 genotype. Residuals after normalization are plotted. Data for cell subsets with sufficient expression for eQTL analysis are shown. T allele is the risk allele for BS.
