# Supplementary material for: Transcriptome analysis of immune cells from Behçet’s syndrome patients: the importance of IL-17-producing cells and antigen-presenting cells in the pathogenesis of Behçet’s syndrome
Source: Arthritis Res Ther. 2022 Aug 8;24:186. doi: 10.1186/s13075-022-02867-x (PMC9358821; doi:10.1186/s13075-022-02867-x)
Supplement: Supplementary file 13 — Additional file 13 eQTL effect of rs4683184 on CCR1, CCR2, CCR3, and CCR5. [file 13075_2022_2867_MOESM13_ESM.pdf]

Additional file 13. eQTL effect of rs4683184 on *CCR1, CCR2, CCR3,* and *CCR5*

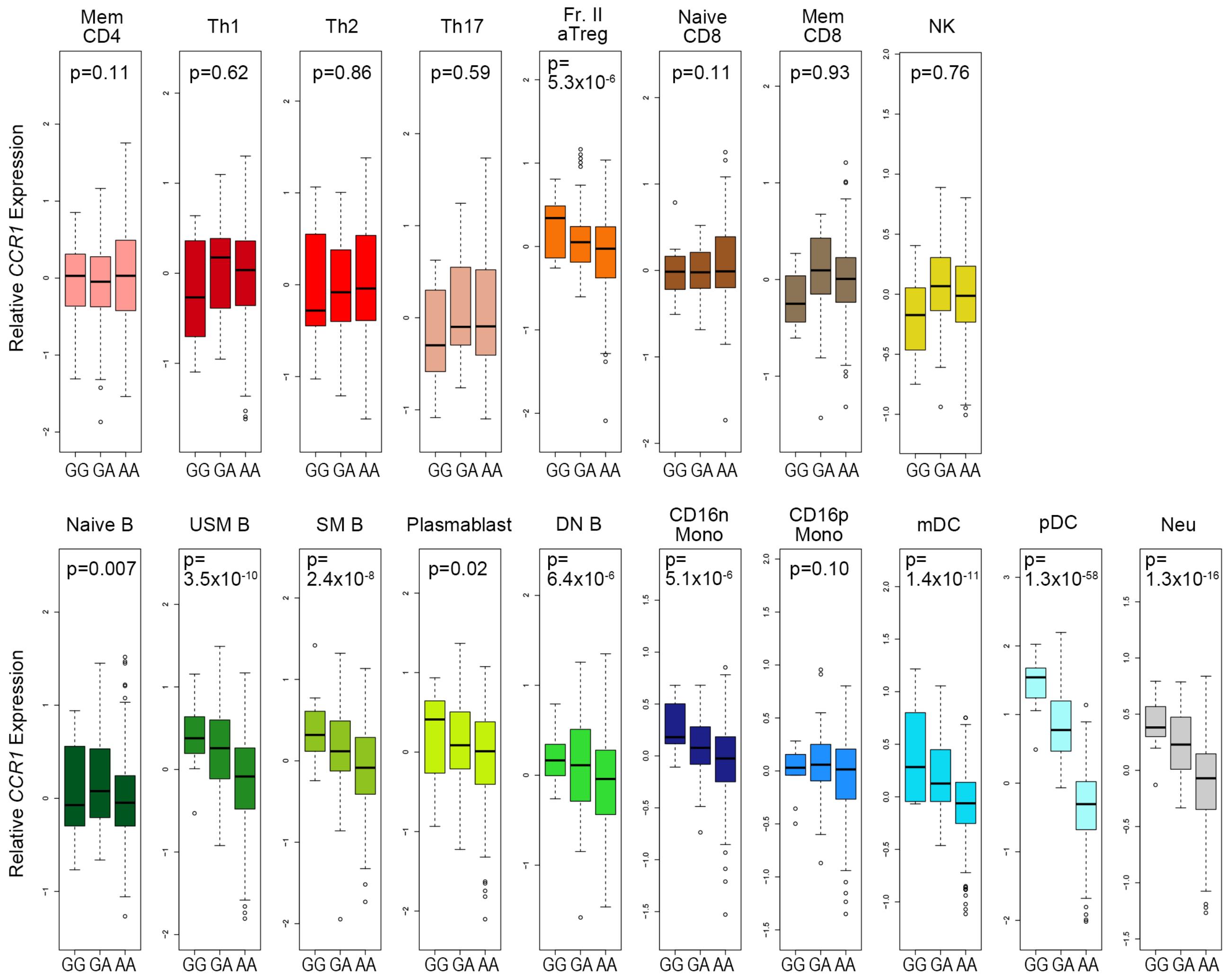

The expression of *CCR1* by rs4683184 genotype. Residuals after normalization are plotted. Data for cell subsets with sufficient expression for eQTL analysis are shown.

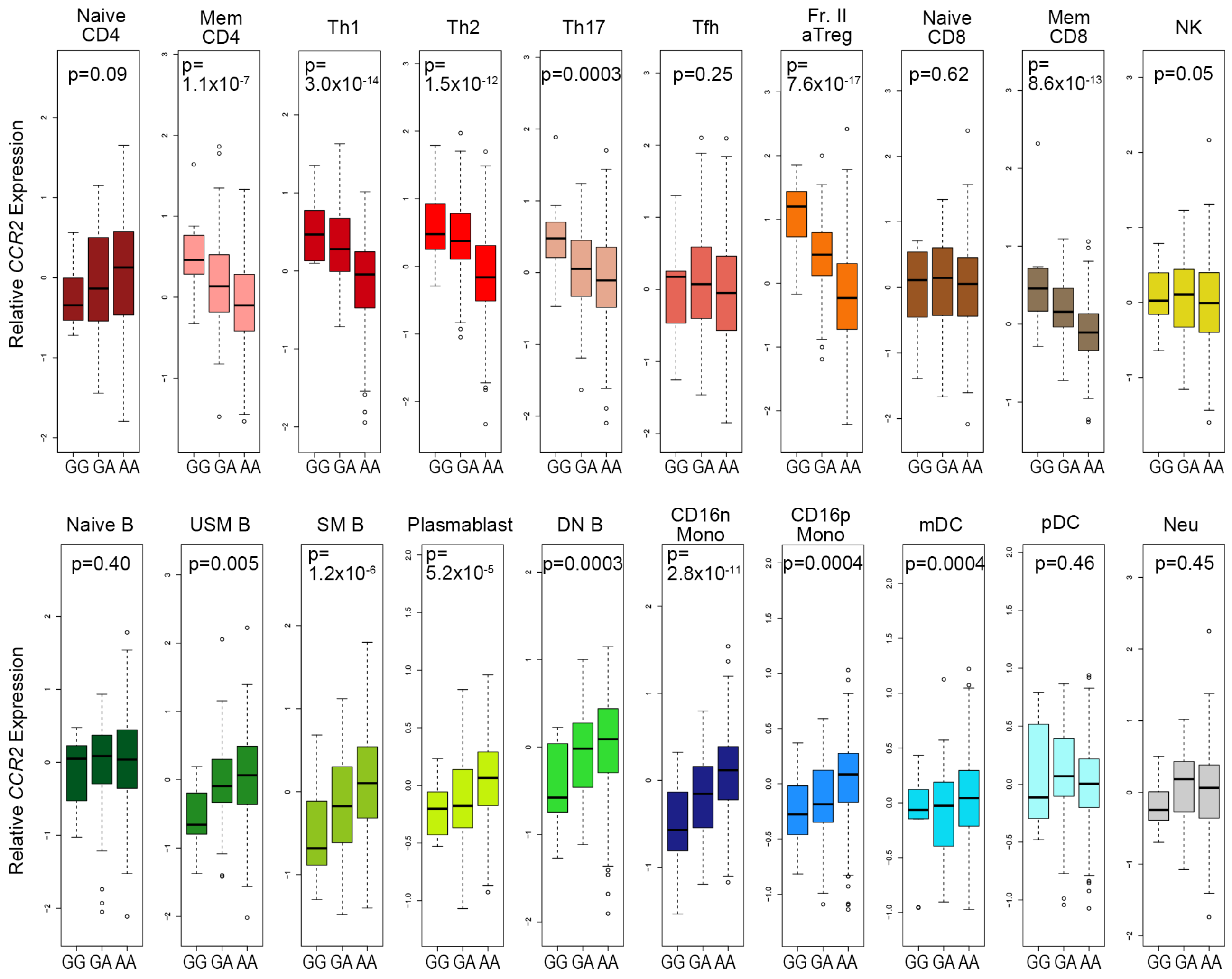

The expression of *CCR2* by rs4683184 genotype. Residuals after normalization are plotted. Data for cell subsets with sufficient expression for eQTL analysis are shown.

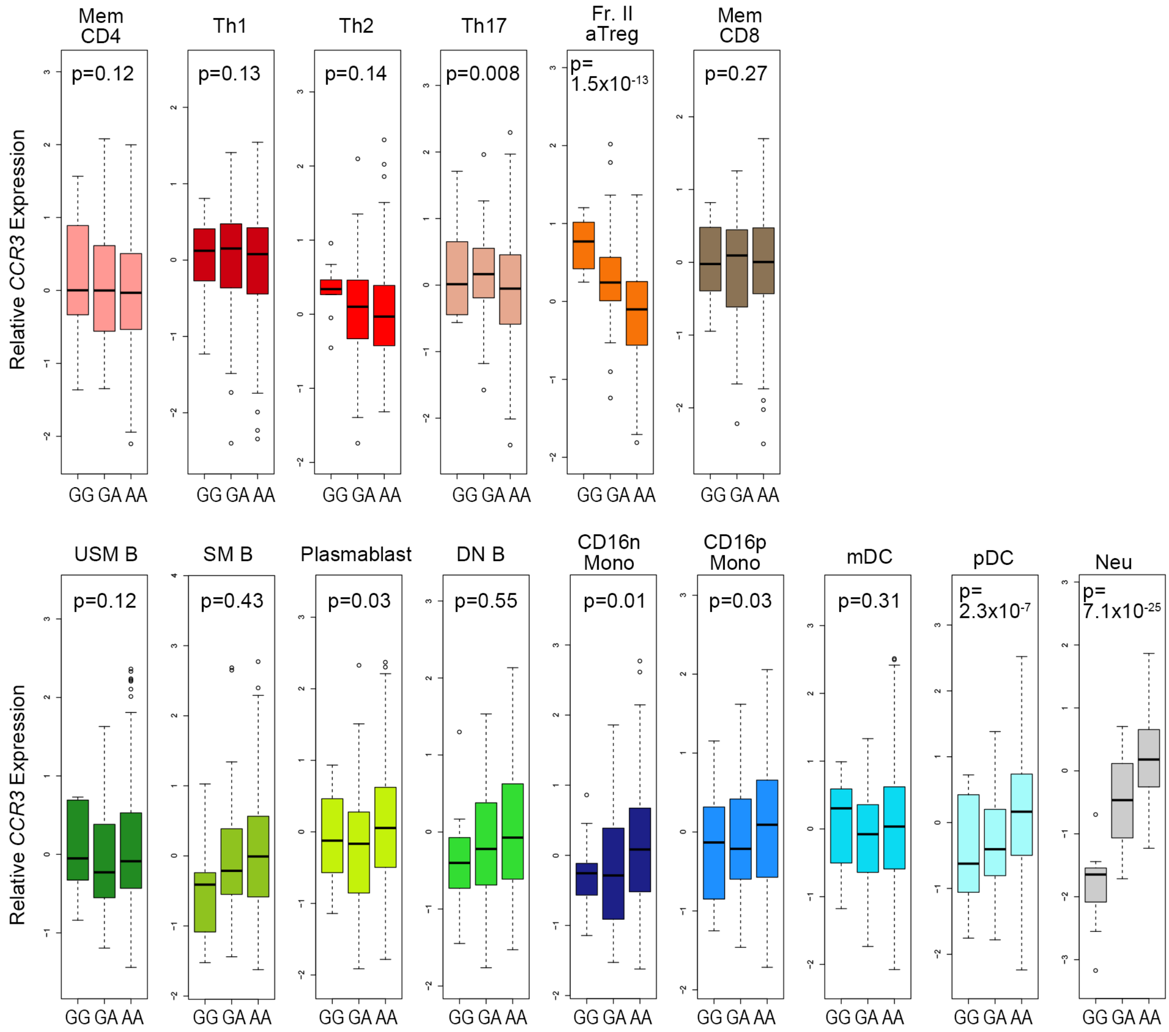

The expression of *CCR3* by rs4683184 genotype. Residuals after normalization are plotted. Data for cell subsets with sufficient expression for eQTL analysis are shown.

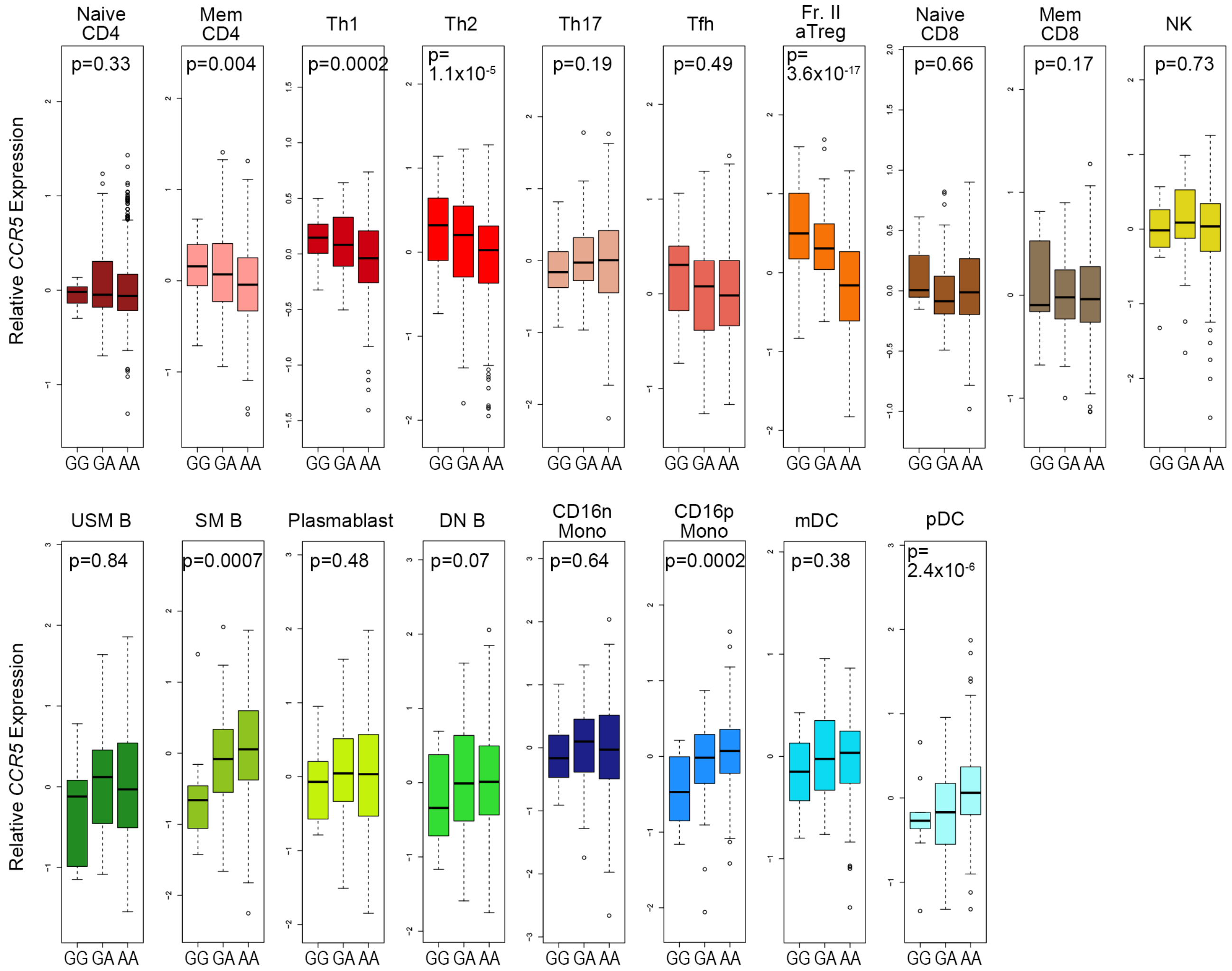

The expression of *CCR5* by rs4683184 genotype. Residuals after normalization are plotted. Data for cell subsets with sufficient expression for eQTL analysis are shown.
