# Supplementary material for: Transcriptome analysis of immune cells from Behçet’s syndrome patients: the importance of IL-17-producing cells and antigen-presenting cells in the pathogenesis of Behçet’s syndrome
Source: Arthritis Res Ther. 2022 Aug 8;24:186. doi: 10.1186/s13075-022-02867-x (PMC9358821; doi:10.1186/s13075-022-02867-x)
Supplement: Supplementary file 14 — Additional file 14 eQTL effect of rs2617170 on KLRK1, KLRC4, and KLRC1. [file 13075_2022_2867_MOESM14_ESM.pdf]

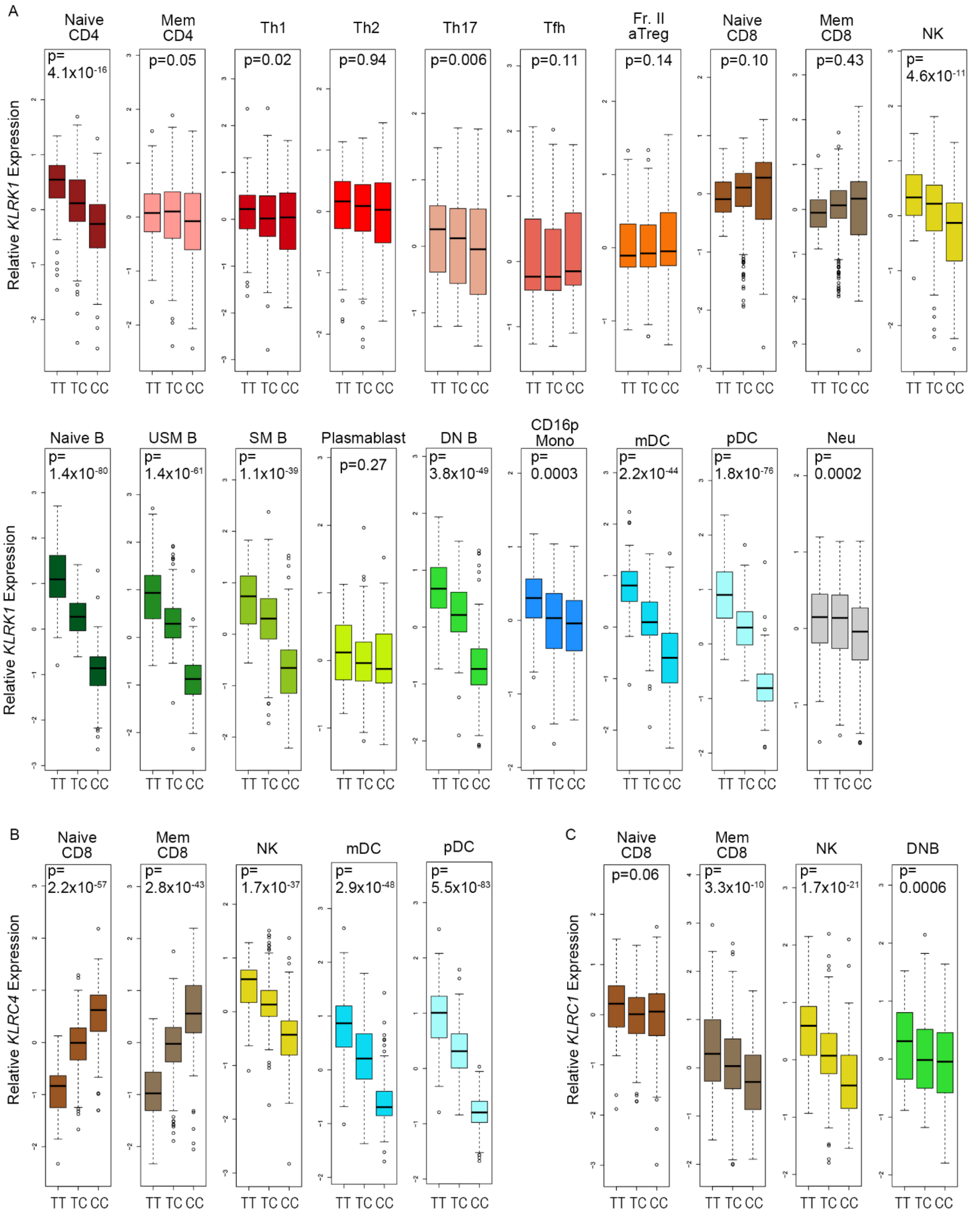

Additional file 14. eQTL effect of rs2617170 on *KLRK1*, *KLRC4*, and *KLRC1*

The expression of *KLRK1* (a), *KLRC4* (b), and *KLRC1* (c) by rs26171470 genotype. Residuals after normalization are plotted.

Data for cell subsets with sufficient expression for eQTL analysis are shown.
