# Supplementary material for: Transcriptome analysis of immune cells from Behçet’s syndrome patients: the importance of IL-17-producing cells and antigen-presenting cells in the pathogenesis of Behçet’s syndrome
Source: Arthritis Res Ther. 2022 Aug 8;24:186. doi: 10.1186/s13075-022-02867-x (PMC9358821; doi:10.1186/s13075-022-02867-x)
Supplement: Supplementary file 15 — Additional file 15. Members of “pDC_15” associated with the diagnosis of BS. [file 13075_2022_2867_MOESM15_ESM.pdf]

Additional file 15. Members of "pDC_15" associated with the diagnosis of BS

Gene	kME	Gene	kME	Gene	kME	Gene	kME
COTL1	0.96	CTSH	0.8	DENND3	0.73	SPECC1	0.69
ABI3	0.94	RIN3	0.8	GSE1	0.73	ZNF366	0.69
IFI30	0.94	CYP2S1	0.79	LGALS3	0.73	ACPP	0.68
CEBPA	0.93	IL1B	0.79	NLRP1	0.73	AIM1	0.68
FGR	0.92	FAM109A	0.78	COL9A2	0.72	FAM179A	0.68
CD22	0.91	FYN	0.78	EMP1	0.72	GNGT2	0.68
CX3CR1	0.91	GSAP	0.78	FAM129A	0.72	KCNE3	0.68
ALOX5	0.9	TSPAN32	0.78	IL7	0.72	KLF8	0.68
BASP1	0.9	ATP1A2	0.77	LY86	0.72	LILRA1	0.68
TIMP1	0.89	BCL6	0.77	STMN2	0.72	MYO1F	0.68
ANXA1	0.88	CD244	0.77	TIAM1	0.72	ZAK	0.68
CEBPD	0.88	IER5	0.77	TM6SF1	0.72	ZNF385A	0.68
CLEC10A	0.88	IGFBP7	0.77	ADAP1	0.71	ANPEP	0.67
ID2	0.88	MS4A7	0.77	ARRB1	0.71	AXL	0.67
SIGLEC1	0.88	PDLIM7	0.77	C19orf38	0.71	IL1RN	0.67
ADAM33	0.87	SCIMP	0.77	CSF3R	0.71	LMNA	0.67
FBLN2	0.87	ASAP1	0.76	GAS7	0.71	NDRG2	0.67
KLF4	0.87	CASP1	0.76	LCP2	0.71	SIGLEC6	0.67
TNFAIP2	0.87	CD200R1	0.76	MPP3	0.71	CD72	0.66
CFP	0.86	CD5	0.76	RTN1	0.71	CFD	0.66
S100A10	0.86	HAMP	0.76	TLR2	0.71	HMOX1	0.66
GLIPR2	0.85	KCNK6	0.76	ASGR2	0.7	ITGB2-AS1	0.66
CSTA	0.84	LST1	0.76	C10orf54	0.7	MRVI1	0.66
LAT2	0.84	PTGS1	0.76	GPAT3	0.7	PHLDA3	0.66
LILRA2	0.84	SPOCK2	0.76	IMPDH1	0.7	TUBA4A	0.66
FGL2	0.83	HAVCR2	0.75	NAIP	0.7	YBX3	0.66
ITGAX	0.83	SPI1	0.75	PPP1R14A	0.7	DOK2	0.65
KCNMB1	0.83	ANXA2	0.74	SLA	0.7	GPR146	0.65
ENHO	0.82	NFAM1	0.74	ADAM28	0.69	LPAR5	0.65
CD300C	0.81	UBASH3B	0.74	ADORA2B	0.69	PSTPIP1	0.65
CD33	0.81	C11orf21	0.73	C20orf27	0.69	VASN	0.65
ITGA5	0.81	CALHM2	0.73	CXCR2	0.69	CSRP1	0.64
CLIC2	0.8	CARD9	0.73	HK1	0.69	GBP2	0.64

(continued on next page)

Gene	kME	Gene	kME	Gene	kME	Gene	kME
HSPA12B	0.64	CD200	0.6	CD2	0.56	AGAP6	0.52
IRF5	0.64	FAM198B	0.6	DHRS3	0.56	ASGR1	0.52
MARVELD1	0.64	NFE2	0.6	FAM20C	0.56	KIAA0922	0.52
MBOAT2	0.64	PSTPIP2	0.6	FAM46C	0.56	LHFP	0.52
P2RY13	0.64	PTPN12	0.6	MYLIP	0.56	NOD1	0.52
PLB1	0.64	SEZ6L	0.6	P2RY2	0.56	OSCAR	0.52
SLC17A9	0.64	TPPP3	0.6	SEMA4A	0.56	PHYHD1	0.52
TSPAN15	0.64	ADAM8	0.59	TMCO4	0.56	PPM1H	0.52
VDR	0.64	CD151	0.59	ASCL2	0.55	RARA-AS1	0.52
CDKN1A	0.63	EFNB1	0.59	LOC642361	0.55	SH2B2	0.52
DENND1A	0.63	MEFV	0.59	MYOM1	0.55	SMOX	0.52
ITGB7	0.63	MYC	0.59	ROBO3	0.55	SPRY2	0.52
LGALS2	0.63	RAB24	0.59	SIGLEC10	0.55	STARD8	0.52
PAK1	0.63	SLC7A7	0.59	UPK3A	0.55	TMEM154	0.52
SH3RF1	0.63	STX11	0.59	AIF1	0.54	ACSF2	0.51
SLC24A4	0.63	ULK2	0.59	CD1C	0.54	FAH	0.51
SYTL1	0.63	CAMP	0.58	CD1D	0.54	FBLN1	0.51
WFDC21P	0.63	CLIP1	0.58	CTSG	0.54	KIT	0.51
APOL3	0.62	KCNE5	0.58	ECE1	0.54	LYZ	0.51
ARHGEF40	0.62	LOC728392	0.58	GYPC	0.54	MFNG	0.51
CPNE2	0.62	LRP1	0.58	LRRFIP1	0.54	NR2F6	0.51
LDLRAP1	0.62	MPST	0.58	MSLN	0.54	RALB	0.51
RXRA	0.62	RGCC	0.58	PIGZ	0.54	CD86	0.5
S100A4	0.62	SLC46A2	0.58	PIK3R6	0.54	FRAT2	0.5
TRERF1	0.62	SNX30	0.58	SEMA4B	0.54	NUDT16P1	0.5
VIPR1	0.62	ZFAND5	0.58	ZDHHC18	0.54	PARP3	0.5
ALDH2	0.61	C17orf85	0.57	ABCB4	0.53	PDLIM1	0.5
ASB2	0.61	HRH2	0.57	ARRDC2	0.53	PIPOX	0.5
ATG16L2	0.61	MOV10	0.57	BAIAP2	0.53	PLXDC2	0.5
MNDA	0.61	NOL4L	0.57	MACROD1	0.53	RNF24	0.5
NUDT16	0.61	PLBD1	0.57	SNHG15	0.53	ZBTB7B	0.5
RAB32	0.61	RAB29	0.57	STAT5A	0.53	ZNF496	0.5
TRAP1	0.61	RHOC	0.57	TCEA3	0.53		
TTC19	0.61	TNFRSF10D	0.57	TMEM173	0.53		
AMPD3	0.6	CAMK1D	0.56	TNFRSF25	0.53		
