# Supplementary material for: Transcriptome analysis of immune cells from Behçet’s syndrome patients: the importance of IL-17-producing cells and antigen-presenting cells in the pathogenesis of Behçet’s syndrome
Source: Arthritis Res Ther. 2022 Aug 8;24:186. doi: 10.1186/s13075-022-02867-x (PMC9358821; doi:10.1186/s13075-022-02867-x)
Supplement: Supplementary file 16 — Additional file 16. Members of “MCD8_08”, the module with strongest positive correlation with HLA-B51 positivity. [file 13075_2022_2867_MOESM16_ESM.pdf]

Additional file 16. Members of "MCD8_08", the module with strongest positive correlation with HLA-B51 positivity

Gene	kME	Gene	kME	Gene	kME	Gene	kME
RORC	0.94	FSD1	0.82	PHACTR2	0.73	SPON1	0.67
CEBPD	0.92	PRSS35	0.82	AMICA1	0.72	ARL14EP	0.66
COLQ	0.92	SPRY1	0.81	CCR2	0.72	CDC42EP3	0.66
SLC4A10	0.92	ADAM12	0.8	SESN1	0.72	FAM43A	0.66
TSPAN15	0.91	CCL20	0.8	CA2	0.71	PDK3	0.66
IL23R	0.9	TRIQK	0.8	CTSH	0.71	RGS2	0.66
SCRN1	0.9	IL17RE	0.79	NMU	0.71	TANC2	0.66
TMIGD2	0.9	KIF5C	0.79	ODF2L	0.71	UBAP1	0.66
CCR6	0.89	NR1D1	0.79	RAB18	0.71	IFNGR1	0.65
KLRB1	0.89	NTN4	0.79	SATB1-AS1	0.71	IL12RB2	0.65
NRIP1	0.89	B3GALT2	0.78	SIGLEC7	0.71	PKDCC	0.65
ABCB1	0.88	BLK	0.78	VLDLR	0.71	PLCB1	0.65
CXXC5	0.88	ELOVL4	0.78	ERICH6-AS1	0.7	RPS6KA3	0.65
FLT4	0.88	IL18R1	0.78	SYTL2	0.7	ARHGAP10	0.64
IL4I1	0.88	DPP4	0.76	TBC1D31	0.7	ROR2	0.64
LTK	0.88	PLEKHA7	0.76	TYROBP	0.7	SLK	0.64
ME1	0.88	SATB1	0.76	IKZF2	0.69	IGF1R	0.63
ZBTB16	0.88	SCART1	0.76	SLAMF1	0.69	PITPNC1	0.63
P2RY14	0.87	TGFA	0.76	UBAC2	0.69	SETD7	0.63
TLE1	0.87	DAB2IP	0.75	DBN1	0.68	CD40LG	0.62
CERK	0.86	TMEM71	0.75	PLD1	0.68	CYB5A	0.62
IL18RAP	0.86	CASC8	0.74	SNX9	0.68	CYTH3	0.62
B3GNT8	0.85	FEZ1	0.74	APOL3	0.67	ELK3	0.62
GPR65	0.84	GALC	0.74	AQP3	0.67	FAM3C	0.62
LL22NC03-75H12.2	0.84	OBFC1	0.74	ATF7IP2	0.67	LBH	0.62
NCR3	0.84	RARG	0.74	HPGD	0.67	SNRK	0.62
CCR1	0.83	COL5A1	0.73	KIT	0.67	ABCF2	0.61
TPBG	0.83	CXCR6	0.73	PDCD4	0.67	CLINT1	0.61
BTBD11	0.82	MATN2	0.73	PLXND1	0.67	MKNK1	0.61

(continued on next page)

Gene	kME	Gene	kME	Gene	kME	Gene	kME
MPZL3	0.61	CRY1	0.56	EXT1	0.53	METTL1	0.5
SIPA1L2	0.61	FHL3	0.56	GYG1	0.53	MYO1D	0.5
KDSR	0.6	IGFBP4	0.56	NUCB2	0.53	RBFOX2	0.5
PELI2	0.6	LONRF3	0.56	PLAC8	0.53	UNC119B	0.5
PLXDC2	0.6	RAB6B	0.56	SPIDR	0.53		
RASGRF2	0.6	RELT	0.56	TANC1	0.53		
UBXN10-AS1	0.6	SPATS2L	0.56	CAP2	0.52		
CAMTA1	0.59	C3AR1	0.55	CDH2	0.52		
CD247	0.59	DSE	0.55	CNN3	0.52		
ERN1	0.59	EDEM2	0.55	CREB3L2	0.52		
FKBP11	0.59	GPR171	0.55	FLI1	0.52		
FOSL2	0.59	LINC00963	0.55	KLRC1	0.52		
KIAA1217	0.59	METTL21B	0.55	LATS2	0.52		
PRNP	0.59	MYBL1	0.55	MBOAT1	0.52		
SPTLC3	0.59	NEO1	0.55	PERP	0.52		
TMEM171	0.59	RUNX2	0.55	PRR29-AS1	0.52		
CPNE7	0.58	SPTSSB	0.55	RHEBL1	0.52		
IL7R	0.58	THEM5	0.55	RRAS2	0.52		
PNP	0.58	TMCC2	0.55	UBXN10	0.52		
ABCD1	0.57	TNFSF13B	0.55	DHX32	0.51		
CREG1	0.57	ZNF880	0.55	PDE4D	0.51		
ITPKA	0.57	DUSP1	0.54	USP43	0.51		
LOC100132111	0.57	LGALS3	0.54	WWC2	0.51		
MICAL2	0.57	LST1	0.54	ADGRA3	0.5		
NCS1	0.57	MPPED2	0.54	ARSJ	0.5		
PROCR	0.57	RIN1	0.54	CREM	0.5		
PTPN13	0.57	CFH	0.53	DAP	0.5		
RAB11FIP1	0.57	DRAXIN	0.53	FCHO2	0.5		
ZNF532	0.57	EAF1	0.53	HIPK2	0.5		
